# Phenotypic diversity and population structure of Pecan (*Carya illinoinensis*) collections reveals geographic patterns

**DOI:** 10.1038/s41598-024-69521-1

**Published:** 2024-08-10

**Authors:** Xinwang Wang, Larry Stein, Mark Black, Keith Kubenka, Jennifer Randall, Chen Ding

**Affiliations:** 1 USDA ARS Pecan Breeding & Genetics, College Station, TX 77845 USA; 2 Texas A&M University AgriLife Research and Extension Center, Uvalde, TX 78802 USA; 3 Entomology, Plant Pathology, and Weed Science, New Mexico State University, Las Cruces, NM 88003 USA; 4 College of Forestry, Wildlife and Environment, Auburn University, Auburn, AL 36849 USA

**Keywords:** Pecan (*Carya illinoinensis*), Genetic diversity, Phenological traits, Population structure, SNP markers, Rootstock breeding, Molecular biology, Plant sciences

## Abstract

Pecan (*Carya illinoinensis*) is an economically important nut crop known for its genetic diversity and adaptability to various climates. Understanding the growth variability, phenological traits, and population structure of pecan populations is crucial for breeding programs and conservation. In this study, plant growth and phenological traits were evaluated over three consecutive seasons (2015–2017) for 550 genotypes from 26 provenances. Significant variations in plant height, stem diameter, and budbreak were observed among provenances, with Southern provenances exhibiting faster growth and earlier budbreak compared to Northern provenances. Population structure analysis using SNP markers revealed eight distinct subpopulations, reflecting genetic differentiation among provenances. Notably, Southern Mexico collections formed two separate clusters, while Western collections, such as 'Allen 3', 'Allen 4', and 'Riverside', were distinguished from others. 'Burkett' and 'Apache' were grouped together due to their shared maternal parentage. Principal component analysis and phylogenetic tree analysis further supported subpopulation differentiation. Genetic differentiation among the 26 populations was evident, with six clusters highly in agreement with the subpopulations identified by STRUCTURE and fastSTRUCTURE. Principal components analysis (PCA) revealed distinct groups, corresponding to subpopulations identified by genetic analysis. Discriminant analysis of PCA (DAPC) based on provenance origin further supported the genetic structure, with clear separation of provenances into distinct clusters. These findings provide valuable insights into the genetic diversity and growth patterns of pecan populations. Understanding the genetic basis of phenological traits and population structure is essential for selecting superior cultivars adapted to diverse environments. The identified subpopulations can guide breeding efforts to develop resilient rootstocks and contribute to the sustainable management of pecan genetic resources. Overall, this study enhances our understanding of pecan genetic diversity and informs conservation and breeding strategies for the long-term viability of pecan cultivation.

## Introduction

The pecan industry has undergone significant changes in recent years, experiencing transformations in production and global markets. The United States of America (USA) used to be the world’s largest pecan producer, but now faces competition from Mexico, which has surpassed the USA, in both acreage and production^1^. Statistical reports in 2023 from International Nut & Dried Fruit Council (INC) showed that Mexico currently accounts for 44% of global pecan production, closely followed by the USA at 40%, with South Africa contributing 10%, and other countries such as China and Brazil comprising the remaining 2%^1^. Between 2013 and 2023, the USA averaged an annual pecan production of 271.4 million pounds, valued at $509.2 million annually^2^. This surge in production can be attributed to the escalating demand for pecan nutmeats, notably from Asian markets, which have been instrumental in driving up prices and fostering expanded cultivation. Notably, China's burgeoning appetite for pecan nutmeats has prompted active efforts to cultivate its pecan industry. These shifts in production and market dynamics are poised to fuel further growth in pecan production globally, including within the USA^1^.

Pecans are distributed from Illinois and Iowa to the Gulf Coast of Louisiana and Texas, marking them as the westernmost species among all *Carya* species^3^. Native pecan populations thrive along rivers across East Texas, with Texas boasting extensive native pecan populations compared to other states^3^. Texas pecan populations in Kinney and Maverick counties blend seamlessly with pecan populations across the Rio Grande in Coahuila, Mexico. Disjunct groups of native pecan populations are also scattered along the southern boundary of Coahuila and in Nuevo Leon^3^. Recent discoveries reveal a wider distribution of native pecan trees in West Texas than previously assumed^3^ (Supplementary Fig. S1).

The United States Department of Agriculture-Agricultural Research Service (USDA-ARS) spearheads the sole national pecan breeding program, aimed at developing pecan scion and rootstock cultivars tailored to specific regions^3–5^. Pecan production in the USA encompasses three primary categories: native, seedling, and improved. While native pecans grow naturally, seedling pecans originate from selected introduced nuts, and improved pecans are selected cultivars grafted onto seedling rootstocks^3,6^. Rootstocks play a pivotal role in commercial pecan orchards, influencing maturation, nut quality, yield, and replanting feasibility^7,8^.

Given the constraints of micropropagation techniques in pecans (*Carya illinoinensis*), growers often turn to locally adapted open-pollinated seeds as rootstock sources. The high heterozygosity and outcrossing traits of open-pollinated pecan populations yield significant diversity across various geographical regions^8,9^. This diversity forms the basis for breeding and selecting rootstocks tailored to local adaptation in diverse growing regions^10–12^. Studies have demonstrated that improved or better-adapted rootstocks can enhance growth, increase production, and improve nut quality, thereby boosting profits for nurserymen and nut producers^13^. Molecular markers play a crucial role in identifying species, geographic regions, and genetic variation within the Carya genus^9,14^. Understanding the genetic structure of pecan germplasm collections is vital for rootstock breeding and association mapping studies, facilitating the identification of genomic regions associated with specific traits^15,16^.

In this study, we established a test orchard by planting a diverse seedling population sourced from open-pollinated seeds of native and cultivar pecans from Mexico and the USA. Our objectives were to evaluate their growth habits in a seedstock test orchard. This orchard serves as a valuable association mapping population, offering unique insights into pecan germplasm diversity. Through field monitoring and next-generation sequencing, we aimed to characterize the variation in growth and phenology among pecan genotypes and elucidate the population structure of open-pollinated seedlings from 26 *C. illinoinensis* collections. This comprehensive analysis provides valuable information for pecan rootstock selection and breeding efforts, ultimately contributing to the sustainability and productivity of commercial orchards for pecan growers.

## Materials and methods

### Pecan materials and experiment design

Open-pollinated seeds (half-sib family) were collected from each of 26 pecan natives and cultivars in Mexico and the USA (https://cgru.usda.gov/carya/) (Supplementary Table S1 and Supplementary Fig. S1). Seeds were germinated in 5.1 cm diameter and 25.4 cm deep Deepots (Stuewe & Sons, Inc., USA), organized in racks of 50 on benches in a greenhouse during the winter of 2012. One-year-old seedlings were then transplanted to an alfalfa field in Uvalde, TX in the spring of 2014, with 10 seedlings per accession planted in a single row within a block arranged in 8 blocks following a complete randomized design.

### Phenotyping

Tree growth vigor can be determined by several different tissues, such as leaves, roots, buds, stem wood, and fruits^17^. Plant height and stem diameter are useful indicators of plant growth vigor. Beginning either in December 2014 or January 2015, plant growth was monitored by measuring plant height and diameter. In the dormant season of 2017, seedling height, in centimeters, was measured using a tape ruler from the ground to the top of the main stem, while stem diameter, in millimeters, was measured using a digital caliper from 2 cm above the ground. Budbreak time, marking the onset of spring growth, was evaluated on April 1, 2015. Budbreak time is a critical event in pecan growth, as it initiates the process of producing new branches, leaves, catkin flowers, and eventually fruits (from current shoots). The timing of budbreak can be influenced by environmental conditions, especially early spring freeze damage in pecans in the southern USA. Terminal-most bud growth (TermBO) was assessed on October 19, 2015. TermBO refers to the bud located at the very tip of the tree’s main trunk. This bud plays a significant role in the growth and development of the stem, as it has the potential to give rise to new shoots, leaves, or flowers. Monitoring the growth and development of the TermBO can provide insights into the tree’s overall health, vigor, and reproductive potential. Both budbreak and TermBO were rated using a standard 1–9 rating scale: 1 = dormant, 2 = swell, 3 = inner scale split, 4 = burst, 5 = first leaflet expansion, 6 = 25% expansion, 7 = 50% leaf expansion, 8 = 75% leaf expansion, 9 = fully expanded leaves^18^. The apical whorl of leaves (ApWhrlO) in a tree refers to a specific arrangement of leaves at the apex or topmost part of the tree’s stem or branch. It is the area where new growth and development occur. The condition or status of ApWhrlO can provide valuable information about the health and vitality of the tree, as well as its stage of growth or maturation. The maturation status of ApWhrlO was rated on October 19, 2015, using a 0–5 scale (0 = none, 1 = early tender, 2 = immature, 3 = mature, 4 = senescent, 5 = dead).

### Sequencing and SNP calls

In the spring of 2017, young, tender leaves were collected from each tree, immediately stored in liquid nitrogen, and then transferred to a − 80 °C freezer. Genomic DNA was extracted and sequenced using the genotyping-by-sequencing (GBS) protocol, following the methods described in the reference^19^. SNP calling was performed using CLC Genomics Workbench V12.0 (Qiagen, Germantown, MD), mapping reads and calling variants against the 87MX3-2.11 ('Oaxaca') V1.0 reference genome^16^. GBS was performed using the protocol in^20^ with the PstI enzyme and with modifications to the requirements to call SNPs that favor the identification of rare alleles and high heterozygosity to utilize the data within the Kinship using GBS with Depth adjustment (KGD) algorithm^21^, which corrects kinship measurements based on the locus read depth. This helps eliminate variations in kinship estimations associated with variable read depths between samples.

To facilitate this, the minimum read depth to call a locus was set to 1, and the minimum proportion of observation of the alternate allele was set to 0.1%. Samples were filtered for those with an average read depth of at least 1 read/locus. SNPs were removed if their average read depth was less than 1, or their Hardy–Weinberg disequilibrium (HWD) (observed frequency of the reference allele homozygote minus its expected value) was less than − 0.05. An HWD lower than − 0.05 tends to indicate multiple loci mapping to the same location in the reference genome.

**Figure 1:**
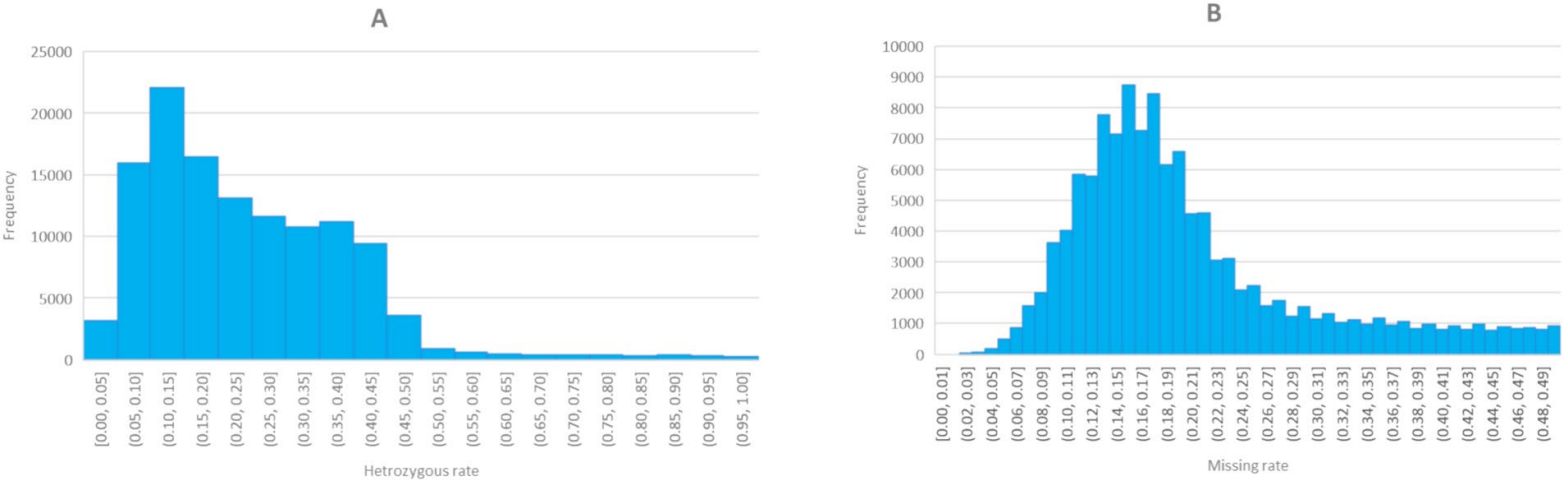
Heterozygous rate (**A**) and missing rate (**B**) of 121,948 SNPs in 550 open-pollinated seedlings om 26 pecan natives and cultivars (*Carya illinoinensis*) from Mexico and the USA, respectively.

**Figure 2:**
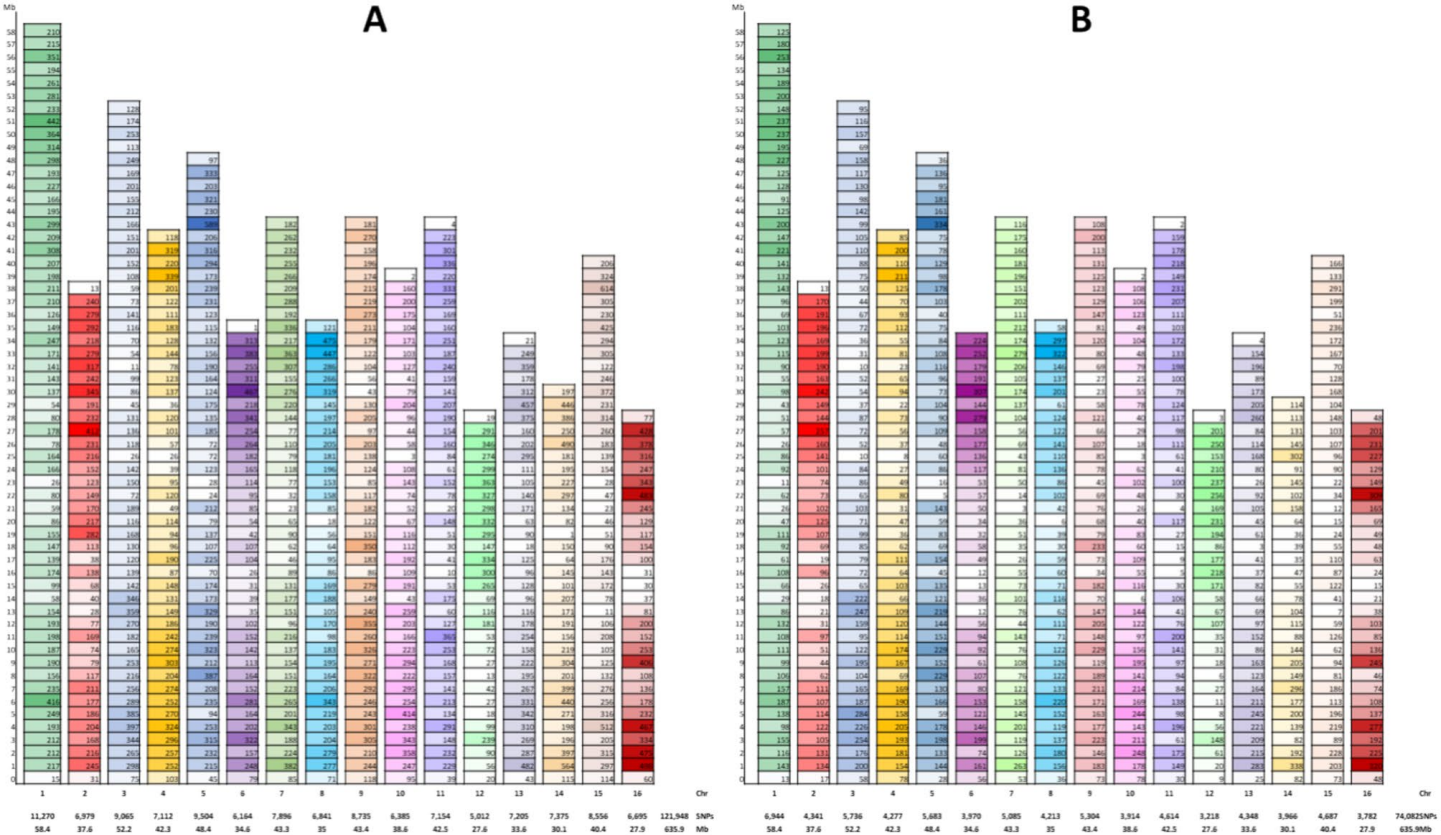
SNPs distributions on 16 chromosomes of *C. illinoinensis*, based on the Illumina reads from 550 open-pollinated seedlings, which were collected from 26 pecan natives and cultivars (*C. illinoinensis*) from Mexico and the USA, respectively. The X-axis shows the chromosomes number, chromosome length (Mb), and total SNPs on each chromosome. The numbers on each chromosome (Y-axis) present the SNP numbers within every 1 Mb. (**A**) Total SNPs were mapped to 16 chromosomes of 87MX3-2.11 (*C. illinoinensis*); (**B**) SNPs were used for diversity analysis.

A total of 272,089 SNPs were generated for the 550 pecan genotypes, with a wide range of frequency of heterozygosity for the individuals (Supplementary Fig. S2A) and the markers (Supplementary Fig. S2B). All SNPs were cleaned using PLINK^22^ to remove SNP markers having (1) multiple alleles and monomorphic alleles; (2) MAF < 0.05 or linkage disequilibrium (LD) > 0.05%; (3) heterozygous rate > 10%; or (4) missing rate higher than 20%. The heterozygous rate (Fig. 1A) and the missing rate (less than 50%) (Fig. 1B) of the remaining 121,948 high-quality SNPs showed a normal distribution across 550 individuals. These SNPs were then mapped to a reference genome of 87MX3-2.11^16^ and assigned onto 16 individual chromosomes. The average map distance between SNP markers varied from 1.65 on chromosome 10–2.45 SNPs on chromosome 14, with an average marker distance of 1.92 SNPs per 10 kb physical interval on the whole genome (Table 1). These SNPs were evenly distributed on the 16 chromosomes throughout the whole genome of *C. illinoinensis*, spanning a total genome size of 635.93 Mb (Fig. 2A). To minimize bias across the individuals, we removed SNPs that had more than a 50% heterozygous rate and more than a 20% missing rate, resulting in 74,082 SNPs. These SNP markers varied from 3218 on chromosome 12 to 6944 on chromosome 1, with an average of 4630 SNPs per chromosome (Fig. 2B). The total length of the SNPs spanned a genome size of 635.76 Mb, with an average mapping distance of 1.17 SNPs per 10 kb (ranging from 1.01 on chromosome 10–1.36 on chromosome 16) (Table 1).

**Table 1:** SNPs on chromosomes of *C. illinoinensis* based on Illumina reads of 550 open-pollinated seedlings from 26 pecan natives and cultivars from Mexico and the USA, respectively.

Chr	Total SNPs mapped on chromosomes				Total SNPs used for GWAS			
	Length (bp)	SNPs	SNPs/10 kb	SNPs/kb	Length (bp)	SNPs	SNPs/10 kb	SNPs/kb
1	5,84,01,512	11,270	1.93	5.18	5,84,01,512	6944	1.19	8.41
2	3,75,57,538	6979	1.86	5.38	3,75,57,538	4341	1.16	8.65
3	5,21,64,292	9065	1.74	5.75	5,21,64,292	5736	1.10	9.09
4	4,23,16,427	7112	1.68	5.95	4,23,16,427	4277	1.01	9.89
5	4,84,36,841	9504	1.96	5.10	4,84,36,841	5683	1.17	8.52
6	3,45,66,641	6164	1.78	5.61	3,44,57,529	3970	1.15	8.68
7	4,33,36,672	7896	1.82	5.49	4,33,36,672	5085	1.17	8.52
8	3,50,27,794	6841	1.95	5.12	3,50,27,794	4213	1.20	8.31
9	4,34,58,297	8735	2.01	4.98	4,34,04,375	5304	1.22	8.18
10	3,85,80,704	6385	1.65	6.04	3,85,80,704	3914	1.01	9.86
11	4,25,23,439	7154	1.68	5.94	4,25,23,439	4614	1.09	9.22
12	2,75,95,804	5012	1.82	5.51	2,75,95,804	3218	1.17	8.58
13	3,35,96,419	7205	2.14	4.66	3,35,96,383	4348	1.29	7.73
14	3,00,84,485	7375	2.45	4.08	3,00,84,485	3966	1.32	7.59
15	4,04,24,760	8556	2.12	4.72	4,04,24,760	4687	1.16	8.62
16	2,78,55,814	6695	2.40	4.16	2,78,55,814	3782	1.36	7.37
Total	63,59,27,439	1,21,948	1.92	5.21	63,57,64,369	74,082	1.17	8.58

### Population structure analysis

Subsequent analysis involved examining population structure using model-based STRUCTURE (Pritchard et al., 2000) and fastSTRUCTURE^23^ programs. STRUCTURE analysis was conducted using a subset of 2713 SNPs for structure analysis by running model-based STRUCTURE. The selected SNPs were transferred into binary data by assigning one homozygote genotype as 0 (for example, AA/TT) and another homozygote genotype (for example, GG/CC) as 1. Heterozygote genotypes were randomly assigned as 0 or 1 considering that these Carya accessions are diploid. For instance, in the case of the heterozygote genotype AT/TA, if A was coded as ‘0’, then T was coded as ‘1’, or vice versa. Similarly, for the heterozygote genotype GC/CG, if G was coded as ‘0’, then T was coded as ‘1’, or vice versa. Genotypes with missing SNPs were coded as − 9. An admixture model with uncorrelated allele frequencies was used to test the subpopulation numbers (K = 2–26). Each K was run five times with a burn-in period of 100,000 steps followed by 1,000,000 Monte Carlo Markov Chain (MCMC) replicates. The 26 pecan genotypes are assumed to be 26 populations with a range of 6–98 individuals. For the determination of the best subpopulation number based on the most likely number of clusters (K), the plateau criterion^24^ and the ΔK method^25^ in Structure Harvester^26^ were used. After knowing the best clusters or sub-populations (K = 8 in this study), another run with 100,000 burn-in and 10,000,000 MCMC for K = 4–13 with 5 reps for each K were conducted on STRUCTURE, to finally determine the best subpopulation numbers. After the STRUCTURE run, the log probability of data [LnP(D)] was estimated for each run, and an ad hoc statistic, ΔK, that was based on the rate of change in LnP(D) between successive K values was used to determine the true number of subpopulations^25^. The best subpopulation was visualized using the Structure Harvester program^26^. Similarly, fastSTRUCTURE analysis utilized a larger set of 11,408 SNPs for exploring population structure. STRUCTURE uses a Bayesian approach to estimate global ancestry but limits it to a small number of markers. However, fastSTRUCTURE is a modern extension of model-based STRUCTURE that can handle a larger number of markers and greatly speed up the inference of population structure while achieving accuracies comparable to STRUCTURE^23^. To better understand the population structure of the 550 pecan genotypes, inferred population structures from two programs were compared. A covariance matrix of each accession was obtained from the inferred subpopulation and used for population genetic diversity analysis.

### Constructing relationship matrices

From the total SNPs that were mapped to 16 chromosomes, we removed SNPs having NN and/or a low call rate < 16% and constructed a genomic relationship matrix (G-matrix) using the R snpReady package (94,250 SNPs in this study), which represents as many different gene loci as possible throughout the whole genome. A pedigree-based relationship matrix (A) was developed utilizing the ASReml-R v.3 package^27^. The realized genomic relationship matrix (G) in the genomic selection (GS) models was computed with the “A.mat” function of the R package rrBLUP^28^ with the default options, which is equivalent to the formula described previously^29^. The correlation coefficient of the A-matrix and the G-matrix was 0.706, indicating that the genomic selection (GS) is efficient using the SNPs selected in this study. All analyses were conducted in the R v.3.6.1 environment^30^, including constructing the marker-based dominance genetic effect matrix, while no dominance relationship matrix was available for the pedigree-based analysis^31^.

### Discriminant analysis of principal components (DAPC)

The overall genetic structure among provenances used was based on SNP frequency of all sampled trees. Discriminant Analysis of Principal Components (DAPC) was conducted using the adegenet package in R^30,31^. DAPC was performed as a DA after the PCA, while prior information of population structure was also used. The inference of the most likely number of clusters was obtained using Bayesian information criterion. DAPC was used to assess the structure of populations based on PCA and DA after using the prior population information^24,32^.

### Ethics statement

All materials used in the present study belong to USDA ARS Pecan Breeding & Genetics Program. Ethical approval was not required for this study. The authors confirm that the experiments research and field studies on plants, including the collection of plant materials in the present study comply with USDA ARS institutional, national, and international guidelines and legislation.

## Results

### Growth and phenological variation among genotypes

Plant growth was evaluated over three consecutive seasons (from 2015 to 2017). Analysis of variance (ANOVA) showed significant variations among the 8 blocks (replications) for plant height, stem diameter, and budbreak across the 550 genotypes of the 26 provenances (Table 2). Details of the R scripts and all variant analyses can be found in Supplementary Table S2. TermBO and ApWhrlO showed no significant differences in the blocks. Genotypes exhibited significant differences for all five traits evaluated. Additionally, genotypes and replications had significant interaction, except for TermBO (Table 2). Among the five traits, plant height, diameter, and budbreak had higher broad-sense heritability (Table 2). TermBO and ApWhrlO presented middle to low heritability (0.59 and 0.36, respectively).

**Table 2:** Analysis of variance (ANOVA) and trait broad-sense heritability.

Trait	Source	Df	Sum Sq	Mean Sq	F value	Pr(> F)	Heritability
Height (cm)	Genotype	25	2,120,678	84,827	25.8120	< 2e−16***	0.7838
	Rep	7	551,714	78,816	23.9830	< 2e−16***	
	Genotype × Rep	156	1,206,921	7737	2.3540	3.91e−15***	
	Residuals	967	3,177,885	3286			
Diameter (mm)	Genotype	25	44,793	1791.7	16.9840	< 2e−16***	0.7105
	Rep	7	11,883	1697.6	16.0910	< 2e−16***	
	Genotype × Rep	156	31,738	203.4	1.9280	2.64e−09***	
	Residuals	967	102,015	105.5			
Budbreak	Genotype	25	799.4	31.97	19.3600	< 2e−16***	0.7724
	Rep	7	50	7.15	4.3290	9.86e−05***	
	Genotype × Rep	156	389.9	2.5	1.5130	0.000163 ***	
	Residuals	967	1597.1	1.65			
TermBO	Genotype	25	51.42	2.0568	9.3800	< 2e−16***	0.5928
	Rep	7	1.03	0.1474	0.6720	0.6960	
	Genotype × Rep	156	22.66	0.1453	0.6630	0.9990	
	Residuals	967	212.04	0.2193			
ApWhrlO	Genotype	25	54.8	2.1938	4.2360	3.26e−11***	0.3559
	Rep	7	5.3	0.7597	1.4670	0.1753	
	Genotype × Rep	156	104.9	0.6724	1.2980	0.0127*	
	Residuals	967	500.8	0.5178			

Significance was indicated as ‘***’ at 0.001, ‘**’ at 0.01, and ‘*’ at 0.05.

The variation of the traits aligned with their provenance origins (Table 3). Overall, Southern provenances exhibited the fastest growth with the tallest plant height, while Northern provenances showed the slowest growth with the lowest plant height. Eastern and Western, including the mixed varieties, showed no significant differences. Seedling stem diameter followed the same growth pattern as plant height (Table 3). Southern provenances had the earliest budbreak time, including those on the top of the main branch (TermBO), and the slowest growth of the apical whorl of leaves at the apex or topmost part of the tree stem or branch (ApWhrlO). Overall, the growth patterns observed over the three conservative seasons after being planted were consistent with expectations. For example, seedstocks from the Southern provenance (Mexico) initiated growth first, such as early bud break, and those from the Northern provenance were the last (Table 3). The Eastern and Western provenances were intermediate, but the natives from the Western provenance were generally faster to begin growth than those from the Eastern provenance, which included the cultivar seedstocks commonly used in the arid regions of Arizona and New Mexico, such as VC1-68 (Table 4).

**Table 3:** Growth and phenological characteristics of pecan seedlings from different geographical origins during the fourth season (2017*) at a field nursery in Uvalde, Texas.

Provenance	Height (cm)			Diameter (mm)			Budbreak			TermBO			ApWhrlO		
	Mean	Std error	Level**	Mean	Std error	Level**	Mean	Std error	Level**	Mean	Std error	Level**	Mean	Std error	Level**
Southern	155.68	7.73	a	27.03	1.30	a	5.10	0.10	a	1.36	0.06	a	2.78	0.06	c
Mix	107.71	4.27	b	21.20	0.70	b	4.72	0.09	a	1.03	0.01	b	3.05	0.04	b
Western	96.08	3.84	bc	19.33	0.67	bc	4.21	0.09	b	1.03	0.01	b	3.13	0.04	ab
Eastern	91.16	3.64	bc	18.04	0.58	c	4.79	0.09	a	1.09	0.03	b	3.00	0.04	b
Northern	73.10	4.52	c	16.42	0.83	c	3.25	0.14	c	1.00	0.00	b	3.30	0.08	a

The term ‘Mix’ indicates that cultivars released by USDA ARS and does not design to any origins in the table. *Seedling height (cm) and stem diameter (mm) was measured in the fourth dormant season (2017), and budbreak, TermBO, and ApWhrlO were rated in the second season (2015). Budbreak and TermBO were rated using 1–9 scale: 1 = dormant, 2 = swell, 3 = inner scale split, 4 = burst, 5 = first leaflet expansion, 6 = 25% expansion, 7 = to 50% leaf expansion, 8 = to 75% leaf expansion, 9 = fully expanded leaves. ApWhrlO was rated using a 0–5 scale (0 = none, 1 = early tender, 2 = immature, 3 = mature, 4 = senescent, 5 = dead). **Levels not connected by same letter are significantly different (p < 0.05) using Tukey–Kramer HSD test.

**Table 4:** Growth and phenological characteristics in an open-pollinated pecan population the fourth season (2017) at a field nursery in Uvalde, Texas.

Accession	Height (cm)			Diameter (mm)			Budbreak			TermBO			ApWhrlO		
	Mean	Std error	Level*	Mean	Std error	Level*	Mean	Std error	Level*	Mean	Std error	Level*	Mean	Std error	Level*
87MX4-5.5	269.79	9.08	a	43.34	1.56	a	5.92	0.18	a	1.92	0.06	a	2.42	0.10	d
VC 1–68	149.73	9.96	b	26.41	1.71	b	5.41	0.20	abcd	1.07	0.07	c	2.70	0.11	cd
San Felipe	138.16	9.96	bc	26.84	1.71	b	4.39	0.20	efg	1.07	0.07	c	3.07	0.11	abc
Shoshoni	122.23	9.17	bcd	24.23	1.57	bc	4.71	0.19	cdef	1.02	0.06	c	3.15	0.10	abc
Frutoso	118.44	8.91	bcde	22.29	1.53	bcd	4.58	0.18	cdef	1.00	0.06	c	3.05	0.10	abc
87MX5-1.7	117.42	10.08	bcde	18.93	1.73	bcdef	4.65	0.20	cdef	1.53	0.07	b	2.63	0.11	cd
A-93	116.28	8.99	bcde	21.61	1.54	bcde	5.44	0.18	abc	1.00	0.06	c	2.85	0.10	bcd
Riverside	114.71	8.33	bcde	21.56	1.43	bcde	4.60	0.17	cdef	1.02	0.06	c	3.06	0.09	abc
87MX1-1.2	111.26	9.08	bcdef	22.21	1.56	bcd	5.17	0.18	abcde	1.04	0.06	c	2.98	0.10	abc
Apache	110.39	8.26	bcdef	21.89	1.42	bcde	4.89	0.17	bcde	1.05	0.06	c	3.20	0.09	abc
Burkett	104.10	10.45	bcdefg	21.98	1.80	bcde	5.05	0.21	abcde	1.03	0.07	c	2.95	0.12	abcd
Baker	101.46	9.17	bcdefg	19.48	1.57	bcdef	5.15	0.19	abcde	1.04	0.06	c	3.02	0.10	abc
Sioux	96.02	10.20	cdefg	19.57	1.75	bcdef	4.40	0.21	defg	1.00	0.07	c	2.88	0.11	abcd
Elliott	92.80	8.99	cdefg	19.56	1.54	bcdef	5.69	0.18	ab	1.15	0.06	c	3.00	0.10	abc
Giles	90.13	9.64	cdefg	19.23	1.66	bcdef	3.83	0.20	fgh	1.00	0.07	c	3.21	0.11	abc
Allen4	84.00	11.87	cdefg	17.16	2.04	cdef	3.52	0.24	ghi	1.06	0.08	c	3.13	0.13	abc
Curtis	82.00	9.44	defg	17.22	1.62	cdef	4.67	0.19	cdef	1.06	0.07	c	3.14	0.11	abc
Stein	79.57	9.75	defg	16.89	1.67	cdef	4.70	0.20	cdef	1.00	0.07	c	3.26	0.11	abc
Moore	73.97	10.72	defg	14.13	1.84	ef	4.47	0.22	cdefg	1.05	0.07	c	3.03	0.12	abc
Ideal	73.50	9.96	defg	14.75	1.71	def	3.07	0.20	hi	1.05	0.07	c	3.30	0.11	ab
Wichita	73.36	9.96	defg	15.32	1.71	def	4.55	0.20	cdefg	1.00	0.07	c	3.16	0.11	abc
97CAT11.3	71.64	9.85	efg	14.49	1.69	ef	2.91	0.20	hi	1.27	0.07	bc	2.96	0.11	abc
Choctaw	70.86	14.42	efg	16.29	2.48	cdef	3.81	0.29	fghi	1.00	0.10	c	3.14	0.16	abc
Major	58.71	11.34	fg	14.35	1.95	ef	2.59	0.23	i	1.00	0.08	c	3.44	0.13	a
Peruque	58.29	14.42	fg	13.48	2.48	ef	3.05	0.29	hi	1.00	0.10	c	3.29	0.16	abc
Allen3	43.13	13.78	g	10.83	2.37	f	3.48	0.28	ghi	1.00	0.10	c	3.22	0.15	abc

The measurements and abbreviations of each trait refer to Table 2. *Levels not connected by same letter are significantly different (p < 0.05) using Tukey–Kramer HSD test.

Among the 26 provenances, 87MX4-5.5 grew the fastest and was the tallest (269.8 cm) than any of the other provenances, followed by VC1-68 (149.7 cm) and Dan Felipe (138.2 cm) (Table 4). Allen 3 exhibited the slowest growth and remained the shortest plant throughout all seasons. Stem diameter followed a similar trend as plant height with 87MX4-5.5 being outstanding in vigor. Notably, after 2017, the seedlings of 87MX4-5.5 continuously showed vigorous growth compared to other provenances (data not shown), presenting a potential rootstock. In comparison, a Southern seedstock, 87MX4-5.5 exhibited the earliest budbreak time and a Northern seedstock ‘Major’ the latest budbreak time (Table 4). Similar patterns were observed for plant growth, budbreak, and TermBO for these 26 pecan collections, indicating a strong association with their geographical origins (Table 3). ApWhrlO showed an opposite trend with budbreak time, with Southern seedstocks (e.g., 87MX4-5.5) being the latest maturation and Northern seedstocks (‘Major’) the earliest maturation (Tables 3 and 4).

### Population structure

**Figure 3:**
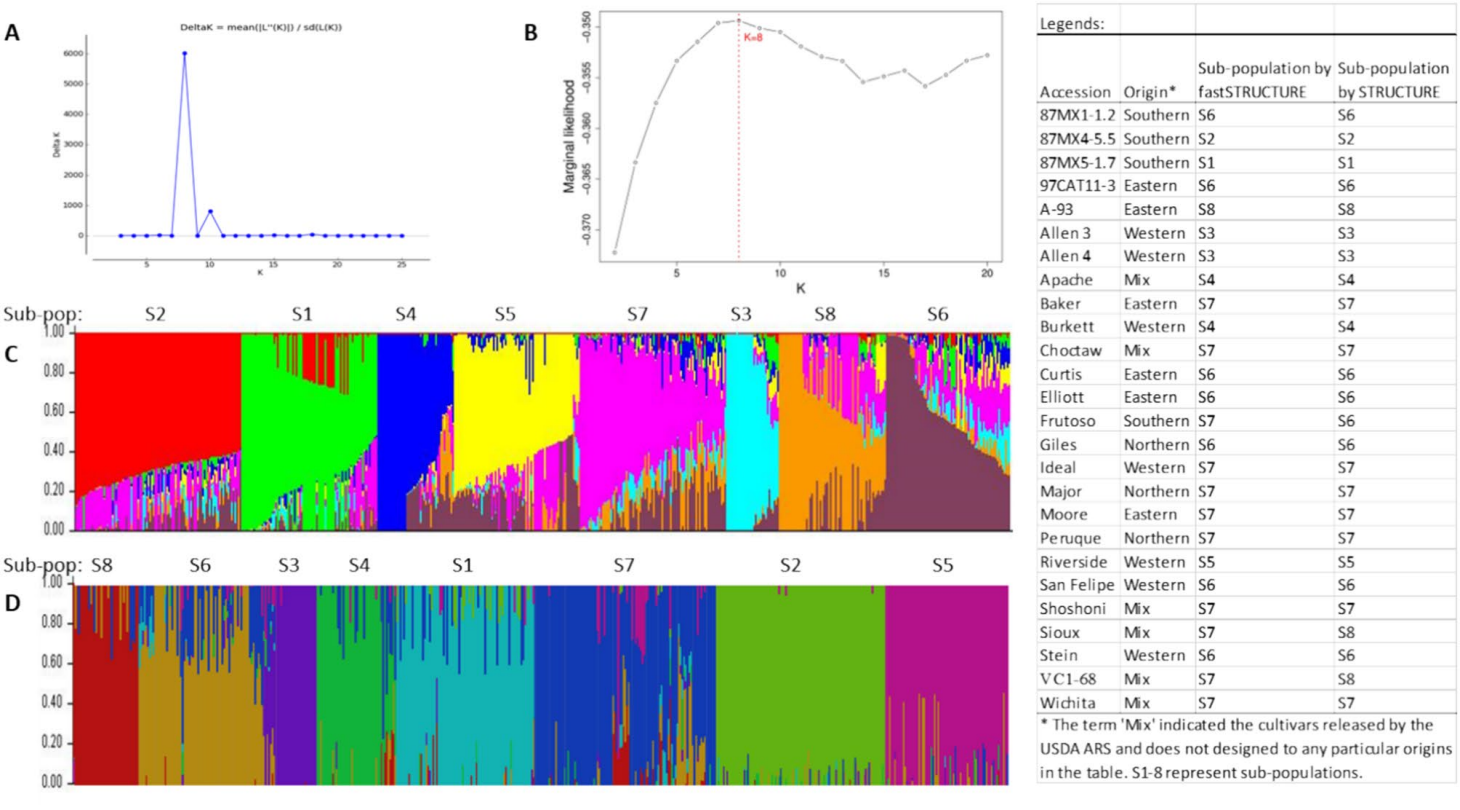
Inferred population structure of 550 open-pollinated seedlings from 26 pecan natives and cultivars (*Carya illinoinensis*) from Mexico and the USA, based on 2713 and 11,408 polymorphic SNPs using STRUCTURE and fastSTRUCTURE, respectively. This figure showed the best subpopulations (K = 8) inferred by STRUCTURE (**A**) and by fastSTRUCTURE (**B**), plots clustered by Q value in STRUCTURE (**C**) and fastSTRUCTURE (**D**), respectively. Each individual is represented by a vertical bar partitioned into k-colored segments, in which the length of each segment represents the estimated membership proportions to each cluster. The vertical bars with the most portion of the same colors are grouped into one cluster. The sub-populations were present on the top of the plot C and D, respectively.

STRUCTURE results for all possible K clusters are shown in Supplementary Fig. S3. When K = 2, two major Mexico seedstocks, 87MX5-1.7 and 87MX4-5.5, were grouped into one cluster, separated from the other seedstocks, which were in a mixed group, including one small Mexico collection, 87MX1-1.2. When K = 3, two Mexico collections were separated from each other, and the other collections were still in a mixed group. When K = 4, three western collections, ‘Allen 3’, ‘Allen 4’, and ‘Riverside’, were distinguished from the mixed group. When K = 5, ‘Riverside’ was alone in one cluster, ‘Allen 3’, ‘Allen 4’, and ‘Burkett’ were in another, and the rest were still in the mixed group. When K = 6, ‘Allen 3’ and ‘Allen 4’ were in one group, ‘Burkett’ and ‘Apache’ were in another, and the rest were in the mixed group. When K = 7, the mixed group was separated into two groups (Supplementary Fig. S4). When K = 8, the ad hoc quantity ΔK shows a clear peak (Figs. 3A and Supplementary Fig. S3), suggesting that eight best subpopulations (designated as S1 to S8) were distinguished from the 26 given populations (Fig. 3C). Subpopulation S1 includes all seedlings of 87MX5-1.7 (80 open-pollinated seedlings), S2 includes 87MX4-5.5 (98), S3 consists of ‘Allen 3’ (7) and ‘Allen 4’ (24), S4 includes ‘Burkett’ (37) and ‘Apache’ (9), S5 contains ‘Riverside’ alone (71), S6 consists of eight collections (87MX1-1.2, ‘97CAT11-3’, ‘Curtis’, ‘Elliott’, ‘Frutoso’, ‘Giles’, ‘San Felipe’, and ‘Stein’) with a total of 81 seedlings, S7 contains 10 collections (‘Baker’, ‘Choctaw’, ‘Ideal’, ‘Major’, ‘Moore’, ‘Peruque’, ‘Shoshoni’, ‘Sioux’, ‘VC1-68’, and ‘Wichita’) with a total of 96 seedlings, and S8 contains ‘A-93’ only (47 open-pollinated seedlings) (Fig. 3B, Supplementary Table S3).

A similar population structure pattern was predicted by fastSTRUCTURE, with 11,408 polymorphic SNPs and K = 8 being the best level of population structure, albeit with minor deviations (Fig. 3B and Supplementary Fig. S4). The subpopulations S1–S6 identified by both programs exhibited significant similarity, as shown in Fig. 3D, and further supported by the analysis depicted in Supplementary Fig. S4. The other two subpopulations, S7–S8, showed discrepancies. Subpopulation S8 in fastSTRUCTURE contained only the A-93 collection (Supplementary Table S4). However, subpopulation S8 in STRUCTURE also contains a portion of ‘Sioux’ and ‘VC1-68’, which had the same level group memberships in subpopulation S7 and S8 in STRUCTURE (Supplementary Table S3). Additionally, the ‘Frutoso’ collection can be clustered in subpopulation S7 but can also be clustered into subpopulation S6 in fastSTRUCTURE due to the close group membership in the two subpopulations (Supplementary Fig. S4). Based on the highest group memberships, the 8 subpopulations inferred by fastSTRUCTURE (Supplementary Table S4) showed no ambiguous population structure compared to those in STRUCTURE (Supplementary Table S3).

Therefore, a K-value of 8 was selected to describe the genetic variation of the 550 open-pollinated seedlings from 26 pecan collections. Of the 8 subpopulations, the S1–S3 subpopulations had lower heterozygosity (average distance) and higher genetic differentiation (Fst), and therefore were clearly separated from each other (Table 5) and distinguished from other collections (S4–S8) because of their geographical regions. 87MX5-1.7 (S1) was from the north of Mexico and 87MX4-5.5 (S2) from the south of Mexico. Surprisingly, another Mexico collection, 87MX1-1.2, was not grouped either in S1 or S2 but instead in subpopulation S6 with some western collections (San Felipe and Stein) (Supplementary Table S1). Both ‘Allen 3’ and ‘Allen 4’ were from the west of Texas and grouped in subpopulation S3. Subpopulation S4 included ‘Apache’ and ‘Burkett’ and was separated from other populations because ‘Burkett’, native to Texas, was one of the parents of ‘Apache’. ‘Riverside’ stood alone in subpopulation S5. It is a commonly used seedstock in California but is native to the southwest of Texas (Grauke, 2019). The individuals of the accessions within subpopulations S6, S7, and S8 were ambiguous. For example, ‘Stein’, ‘San Felipe’, ‘97CAT11-3’, and ‘Curtis’ were clustered in S6 but also had a higher portion of group membership with the seedlings in subpopulation S7 (Supplementary Table S3). ‘Baker’, ‘Moore’, and ‘Wichita’ in S7 also had a higher portion of group membership in S6 and/or S8. ‘Sioux’ and ‘VC1-68’ in S8 had a higher portion of group membership in S6 and/or S7.

**Table 5:** Expected heterozygosity in the 8 subpopulations of the 550 individuals of 26 pecan natives and cultivars in *C. illinoinensis* species from Mexico and the USA in the STRUCTURE.

Subpopulation	Expected heterozygosity	Genetic differentiation (*F*_*st*_)
S1	0.2033	0.3813
S2	0.2001	0.4143
S3	0.2140	0.3451
S4	0.2183	0.2874
S5	0.2025	0.3242
S6	0.2225	0.1681
S7	0.2398	0.1777
S8	0.2149	0.2973

### Genetic differentiation

**Figure 4:**
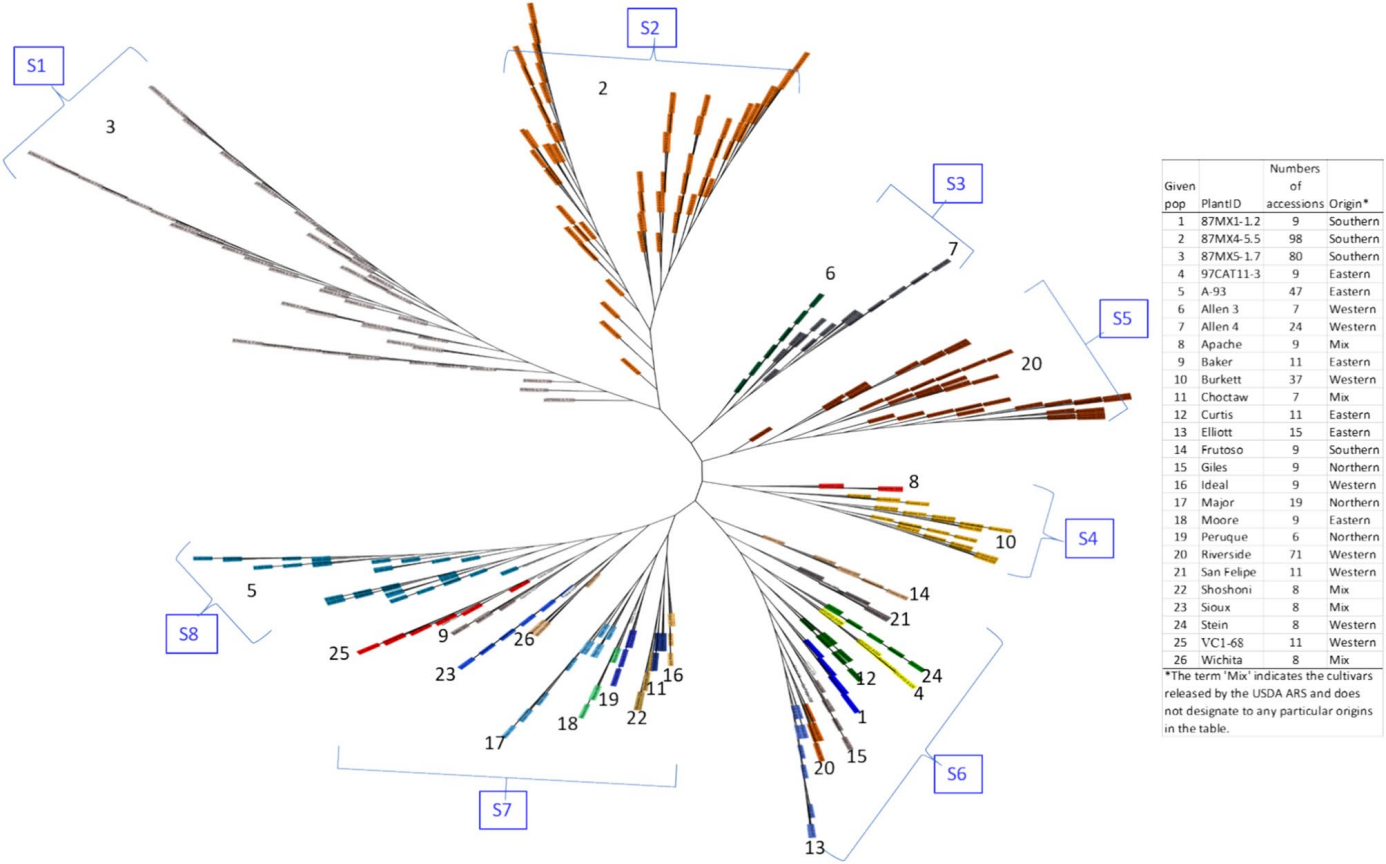
UPGMA unrooted tree of 550 open-pollinated seedlings from 26 pecan natives and cultivars (*Carya illinoinensis*) from Mexico and the USA. Each genotype was separated by different colors.

The genetic differentiation of the 26 given populations was further assessed through a phylogenetic tree (Fig. 4). The results showed distinct differentiation among the 26 populations, with six clusters (S1–S6) highly in agreement with the S1–S6 in both STRUCTURE (Fig. 3C) and fastSTRUCTURE (Fig. 3D). The remaining genotypes could be clustered into two groups, either in agreement with the S7 and S8 in STRUCTURE (Fig. 3C, Supplementary Table S3) or the S7 and S8 in fastSTRUCTURE (Fig. 3D, Supplementary Table S4).

**Figure 5:**
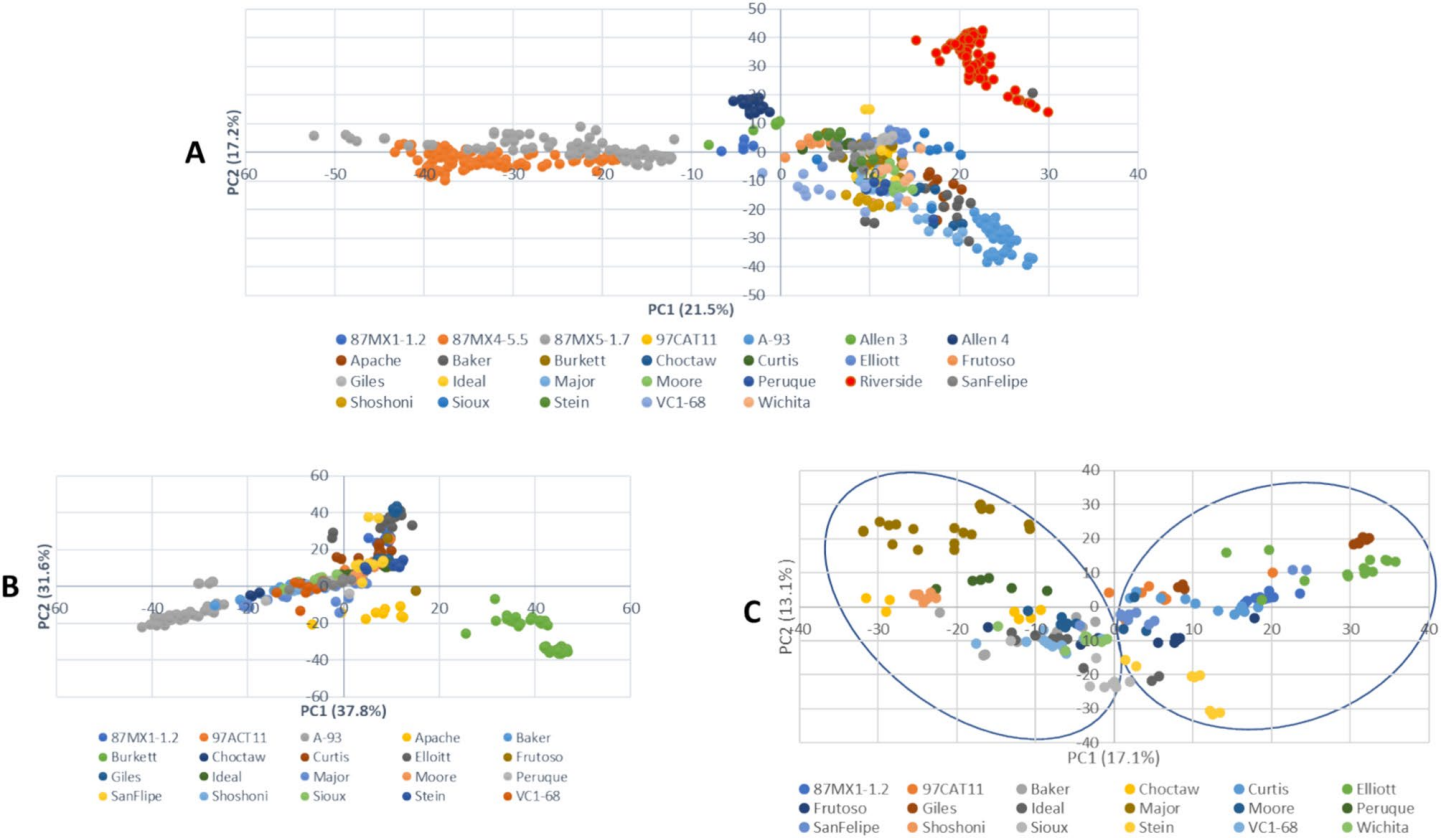
Plots of two principal components of the 26 putative populations (*Carya illinoinensis*) with 74,082 SNPs. (**A**) Plots of the 550 open-pollinated seedlings of the 26 putative populations. (**B**) Plots of 270 open-pollinated seedlings of 21 populations [excluded 87MX5-1.7, 87MX4-5.5, Allen 3, Allen 4, and Riverside which were distinct from the mix group in plot (**A**)]; (**C**) plots of 177 open-pollinated seedlings of 18 populations [excluded Burkett, Apache and A-93 that were distinct from the mix population in plot (**B**)].

Principal component analysis (PCA) was conducted in TASSEL to plot the first two principal components reflecting population variation among the 26 given populations. The plot of PC1 (18.8% of variance) and PC2 (14.6% of variance) revealed four distinct groups (87MX5-1.7, 87MX4-5.5, ‘Allen 3’ and ‘Allen 4’, and ‘Riverside’) that were distinguished from other genotypes (the rest of the 21 genotypes were in one group), which correspondingly agrees with the S1, S2, S3, and S5 subpopulations in STRUCTURE and fastSTRUCTURE (Fig. 5A). Therefore, we excluded these five populations and conducted PCA analysis for the remaining 21 populations (Fig. 5B,C). From the PCA plots of the 270 individuals from those 21 populations, ‘Burkett’ and ‘Apache’ clustered into one group and ‘A-93’ in another group (Fig. 5B), which fell into S4 and S8 in STRUCTURE and fastSTRUCTURE, respectively (Fig. 3). When ‘Burkett’, ‘Apache’, and ‘A-93’ were excluded, the PCA plots of the rest of the 177 individuals from 18 populations showed two adjacent subpopulations (Fig. 5C), which matches S6 and S7 respectively in STRUCTURE and fastSTRUCTURE (Fig. 3).

**Figure 6:**
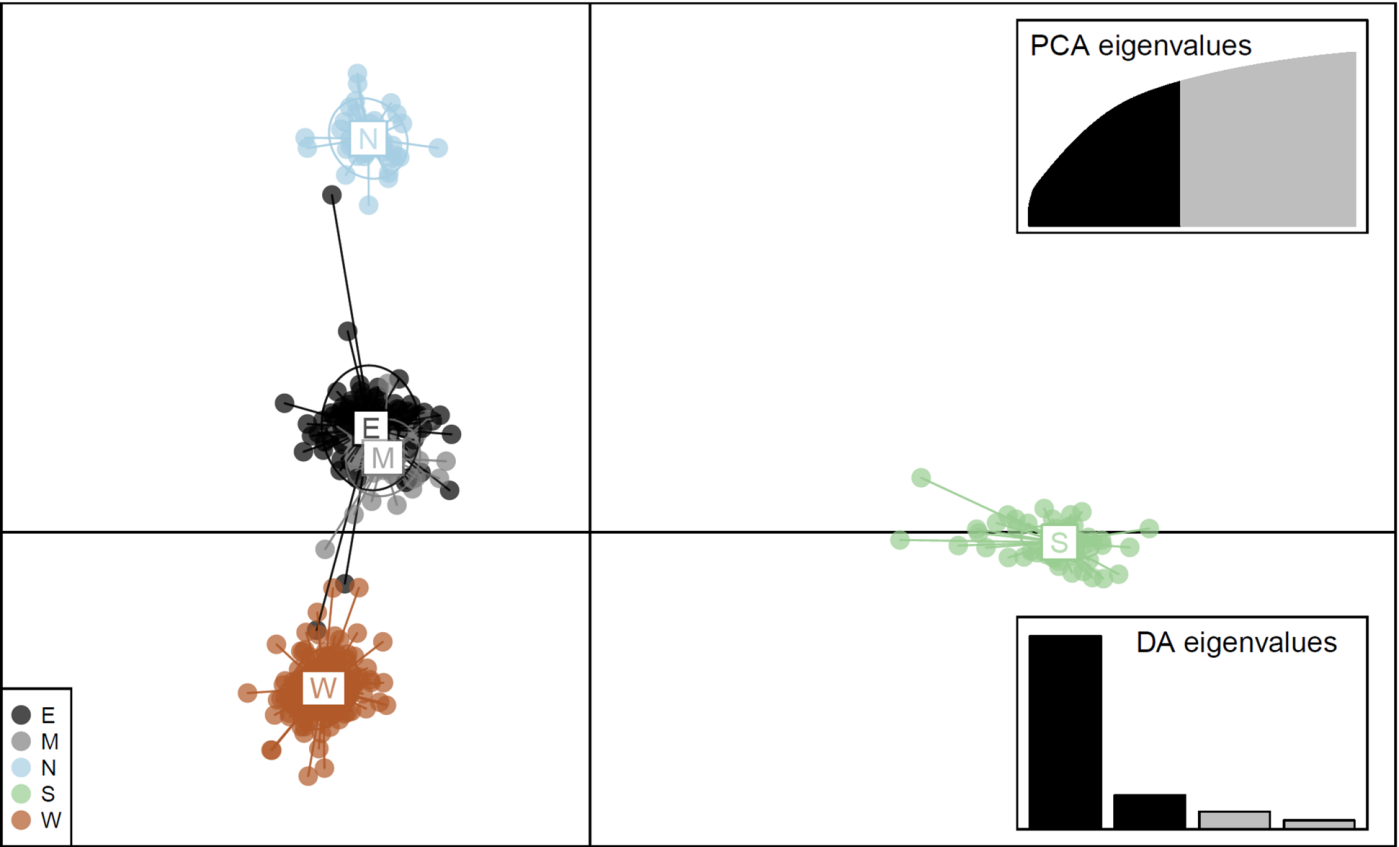
Scatterplot from discriminant analysis of principal components (DAPC) of the first two principal components by geographical origins. *E* Eastern, *S* Southern, *W* Western, *N* Northern, *M* mix.

When the population structure was clustered based on the provenance origin of the 26 collections using DAPC (instead of PCA), the provenances were clearly separated (Fig. 6). The first PC (x-axis) distinguished the provenance S from the rest of the provenances, and the second PC had three groups, i.e., W, N, and M/E (i.e. Mix and E together) provenances so that four DA clusters were demonstrated. This genomic pattern generally agreed with the previous population structures (Figs. 4 and 5).

## Discussion

### Open-pollinated seed collection

In this evaluation, 26 collections were included, consisting of various seedstocks commonly used in the western pecan industry. Two standard seedstocks, ‘Burket’ and ‘Riverside’, traditionally utilized in California but native to Texas (Supplementary Table S1), performed exceptionally well based on their fast growth and local adaptation and were comparable to other tested seedstocks^5,11,13,33^. Two southern provenance accessions from Mexico, 87MX4-5.5 and 87MX5-1.7, exhibited outstanding vigor but were susceptible to phenological changes in this test and USDA repository orchard (data not shown). The northern provenance collection, ‘Major’, exhibited late growth, early leaf dropping, and reduced tree size. ‘Elliott' is an eastern provenance collection and has been widely used as rootstock in most pecan growth regions. ‘Allen 3’ and ‘Allen 4’ were sourced from western Texas. Given that ‘Burkett’ was one of the parents of ‘Apache’, it is logical to group ‘Allen 3’ and ‘Allen 4’ with ‘Burkett’ and ‘Apache’, respectively. The genetic structure of S6–S8 exhibited a mixture for some pecan cultivars. ‘Curtis’ and ‘Moore’, commonly used seedstocks in the southeastern (Georgia, Alabama, Mississippi, Louisiana, and Oklahoma), actually originated from Florida^34^. However, pecans are not native to Florida, and the movement of pecan nuts by humans has altered their geographical origin, resulting in a reduced genetic distance between them. Statistical analysis of their phenotypic traits revealed significant differences (Table 4).

The observation of budbreak in their second season (2015) showed considerable variation in field observations and statistical analysis. Of the five phenotypic traits, plant height, stem diameter, and budbreak time showed higher broad-sense heritability, which captured their phenotypic variation in the ANOVA (Table 2). In the context of plant breeding, non-additive marker effects may account for a significant proportion of the variation in complex traits, such as plant height, root length, root diameter, and root surface area^35^. Therefore, plant height, diameter, and budbreak can be important indicators for breeding better rootstocks.

### SNP variation and genetic diversity

Pecan nuts are characterized by open-pollination, having a high degree of heterozygosity^36^, and the pecan tree has a lengthy life cycle. These pose a significant challenge in pecan genetic studies because it could take several years of continuous breeding and a lengthy selection process to stabilize the desirable alleles. Through Illumina data analysis of the entire collections, we identified over 270,000 SNPs. Following filtering, we successfully mapped 121,948 high-quality and polymorphic SNPs onto the 16 chromosomes of the reference 87MX3-2.11 genome^16^.

In the present study, most SNP markers (80.8% or 98,550 out of 121,948 SNPs) displayed 10–50% heterozygous alleles across the 550 open-pollinated individuals (Supplementary Fig. S2B). Additionally, 15.6% (19,008 out of 121,948 SNPs) exhibited less than 10% heterozygous alleles, while 3.6% (4390 out of 121,948 SNPs) showed more than 50% heterozygous alleles (Supplementary Fig. S2A). Among the 550 open-pollinated individuals, more than half (52.9% or 291 out of 550 individuals) exhibited 30% heterozygosity, with 1.3% displaying over 30% heterozygosity. Only 15.3% of individuals had less than 10% heterozygosity (Supplementary Fig. S2A). The heterozygosity observed in individuals within a population resulting from natural or controlled crossings contributes to dynamic genetic variations in regions associated with complex traits. It is important to note that a higher rate of heterozygous alleles within the population can introduce bias in model-based STRUCTURE analysis. Therefore, considering the pecan genome's inherent heterozygosity, we removed over 50% of the heterozygous alleles within the population. This step aimed to mitigate the bias generated in model-based clustering methods in STRUCTURE and its extension fastSTRUCTURE, as well as facilitate comparison with phylogenetic tree analysis (implemented in TASSEL). Although the group membership determined by the three methods exhibited some variations, the classification of most individuals within a specific population remained consistent and aligned with their geographical origins.

It is worth noting that heterogeneous and highly heterozygous individuals can affect the accuracy of SNP calls in comparison to homozygous or doubled haploid (DH) samples^37,38^. The presence of a significant proportion of such individuals within a population adds complexity to population structure analysis^15^. Despite efforts to filter out over 50% of heterozygous and low minor allele frequency (MAF) alleles to improve the estimation of relatedness among individuals within and between populations, we still observed some individuals being assigned awkwardly among subpopulations, seemingly conflicting with the geographical regions of their mother trees. This discrepancy can be attributed to unknown pollen sources, non-Mendelian inheritance in certain regions across the 26 pecan collections, or potential sequencing or genotyping errors^21^. On the other hand, the size of a population also plays a role in genetic diversity and population structure^15^. In this study, 20 out of the 26 populations consisted of fewer than 20 open-pollinated individuals. The limited number of individuals in these populations results in reduced genetic variation, making it challenging to differentiate or cluster them into distinct groups. We analyzed 18 of these 20 populations separately (excluding Allen 3, which easily grouped with Allen 4, and 'Apache', which grouped with 'Burket'). However, no significant groups could be distinguished from each other, although it can be assumed that there are two groups (Fig. 5C). While advancements in sequencing technology have allowed for the development of SNP markers on a larger scale and at lower costs, a larger effective population size would be necessary to accurately predict genetic distances and assess the genetic diversity of individuals^39,40^. Including a greater number of individuals is likely to improve allele frequency estimates for different groups, particularly in diversity studies utilizing GBS data^21^.

### Genetic kinship identifies the pollen source of the collection

A southern collection from Mexico, 87MX4-5.5, exhibited rapid growth with taller plant height and larger trunk diameter compared to all other collections in the test (Table 4). The mother tree of 87MX4-5.5 and another Mexico provenance, 87MX5-1.7, are situated next to each other in our repository orchard where the seeds were collected and exhibit complementary bloom patterns. 87MX4-5.5 is a medium-sized tree with a spreading canopy, while 87MX5-1.7 is a tall tree with a vertical canopy. These two Mexico provenances displayed distinct characteristics from the other collections in the structure analysis, maintaining their separation until K = 26. It is possible that pollen from 87MX5-1.7 pollinated the nuts of 87MX4-5.5, contributing to their vigor. Genotype by sequencing (GBS)^19,41^ was utilized in this study to confirm the parentage of the seedlings and assess the measured vigor of 87MX4-5.5 seedlings in relation to their pollination by 87MX5-1.7, based on the kinship between these two seedling populations (Supplementary Figs. S5 and S6). This information provides guidance for selecting suitable seedstocks by using open-pollinated seeds, indicating that candidate seedstock trees should be planted near robust pecan trees that exhibit vigorous growth habits and potentially share the same adaptation region provenance.

### Population structure and geographical region

Gene-flow typically decreases as geographical distance increases^37^. Consequently, genetic divergence in a natural population can be observed through the measurement of genetic distance, enabling the differentiation of collections based on their geographical distances. In this study, the 26 pecan natives and cultivars (*C. illinoinensis*) represent a wide range of geographical regions, spanning from east (Florida) to west (California/Arizona) and from north (Missouri) to south (Mexico) (Supplementary Table S1 and Supplementary Fig. S1). Despite the seeds being collected from mother trees maintained in the USDA provenance orchard in Somerville, TX, the field performance of the open-pollinated seedlings varied significantly based on their geographical regions.

Using a subset of SNPs, the 26 entries exhibited consistent grouping patterns at K = 8 (Fig. 3A). This grouping pattern also aligned with the clusters observed in the phylogenetic or archaeopteryx tree (UPGMA) when all SNP data were utilized in the TASSEL program, with minor discrepancies in accession assignment (Fig. 4). These results suggest that a core set of 2713 SNPs was sufficient to categorize the 26 given populations into 8 subpopulations, capturing their genetic diversity across geographical regions.

Seedlings from two southern provenance collections, 87MX4-5.5 and 87MX5-1.7, along with one western provenance collection, 'Riverside', formed three distinct subpopulations (S1, S2, and S5, respectively, as shown in Fig. 3B), and were clearly separate from other accessions (Figs. 4 and 5). Two closely related accessions from West Texas provenance collections, Allen 3 and Allen 4, were grouped together in one subpopulation (S3) across the three clustering methods (Figs. 3B, 4) due to their shared geographical regions (Supplementary Table S1). 'Burkett' and 'Apache' were assigned to a single subpopulation (S4, Fig. 3B) since ‘Burkett' served as the maternal parent of 'Apache'^3^. This distinctive subpopulation was also evident in the PCA analysis (Fig. 4).

Minor discrepancies in accession assignments were observed in the remaining entries across the different programs (Figs. 3B, 4). Despite their varied geographical regions, distinguishing the open-pollinated progeny of these entries is challenging due to their relatively close geographical distances. Another contributing factor is the limited population size of some entries, with most of them consisting of fewer than 20 open-pollinated seedlings, which may not accurately capture the true variation within each population.

Although the assignment of provenance post-genomic verification was necessary here, population differentiation demonstrated significant clusters based on the experimental design and sampling scheme. Therefore, increasing the population size of each entry in the current *C. illinoinensis* collection is necessary. A comprehensive understanding of the structure of the breeding population or germplasm collection is particularly crucial for scion and rootstock breeding or selection, given the species’ long-life cycle and extensive diversity^13,33,42^.

The UPGMA hierarchical clustering analysis generally supported the subdivision of the population into 8 subpopulations. Specifically, the seedlings within clusters S1, S2, S3, and S4 showed consistent assignment across all three analyses. All 71 seedlings from the 'Riverside' collection were exclusively grouped into subpopulation S5. However, within the 'Riverside' collection, 63 seedlings originating from one mother tree were assigned to group S5, while 8 seedlings from another mother tree in the same orchard were assigned to group S6 (Fig. 4), indicating the influence of pollen sources on genetic diversity.

The remaining three clusters, consisting of 9 of the 26 collections and 8 individuals from 'Riverside', exhibited slight discrepancies among the three clustering methods. This discrepancy is likely due to the small number of individuals within each collection, with a total of 224 individuals ranging from 6 to 15, plus 47 from A-93. To ensure accurate genetic diversity analysis, a larger sample size is essential to reduce SNP calling and genotyping errors^9,14,43^.

A comprehensive understanding of the genetic structure within pecan germplasm collections is crucial for effective breeding and selection strategies. It enables researchers to identify and utilize diverse genetic resources for targeted trait improvement. By analyzing the allelic patterns associated with geographical regions, researchers can gain insights into natural adaptation and the genetic diversity present in pecan populations. Subpopulation identification allows researchers to account for population structure and minimize the risk of false associations when studying complex traits.

Leveraging the genetic diversity present in pecan germplasm collections empowers breeders to make decisions regarding the selection and development of superior rootstocks and scion varieties. This approach allows for the maximization of genetic diversity and enhances the potential gains from selection using genetic tools. In addition to facilitating breeding efforts, understanding the genetic structure of pecan populations also contributes to conservation and sustainable management practices. It plays a crucial role in preserving the natural diversity within the species and ensuring the maintenance of valuable genetic resources for future generations. The knowledge gained from studying the genetic structure of pecan populations provides a foundation for implementing conservation strategies and sustainable management practices. It allows for the identification of key populations and genetic resources that are of particular importance for maintaining species resilience and adaptation in the face of environmental challenges. Furthermore, this understanding can guide the establishment of ex situ conservation programs, such as seed banks or living collections, to safeguard the genetic diversity of pecans for future use.

## Conclusion

This study provides valuable insights into the growth vigor, phenological traits, and genetic diversity of pecan populations across diverse geographic regions. Through field phenotyping and genomic analyses, we found significant variations in growth patterns among provenances, emphasizing the profound influence of environmental factors on pecan phenotypic performance. These findings illustrated the importance of considering geographic origin in breeding programs aimed at developing rootstocks adapted to specific climatic conditions and soil types. Furthermore, the identification of eight distinct subpopulations through population structure analysis offers valuable guidance for conservation efforts and targeted rootstock breeding strategies. From the genetic clusters based on shared ancestry and geographic distribution, we can prioritize germplasm collections that possess unique genetic traits essential for enhancing pecan resilience and productivity. Moreover, this study contributes to our understanding of the evolutionary processes shaping pecan diversity and highlights the need for continued research to unravel the genetic basis of key agronomic traits. By integrating cutting-edge genomic tools with traditional breeding approaches, we can accelerate the development of resilient and sustainable rootstock varieties capable of thriving in diverse agroecosystems. This research benefits pecan growers and breeders for sustainable pecan production by selection and breeding superior rootstocks. Further exploration of the molecular mechanisms underlying pecan adaptation will be crucial for developing innovative breeding strategies and ensuring the long-term sustainability of pecan cultivation. Overall, this study advances our understanding of pecan genetic diversity, enabling the development of cultivars better adapted to diverse environments and contributing to the sustainable management of pecan genetic resources for future generations.

### Supplementary Information

Supplementary Information.

## Data Availability

Details of all 26 collections are available at https://cgru.usda.gov/carya/. The datasets used and/or analyzed during the current study are included in the article/Supplementary files. Sequence and SNP data are available from the corresponding author on reasonable request.
