# Supplementary material for: Phenotypic diversity and population structure of Pecan (Carya illinoinensis) collections reveals geographic patterns
Source: Sci Rep. 2024 Aug 10;14:18592. doi: 10.1038/s41598-024-69521-1 (PMC11316781; doi:10.1038/s41598-024-69521-1)
Supplement: Supplementary file 1 — Supplementary Information. [file 41598_2024_69521_MOESM1_ESM.zip › Suppl Table S2 R scripts and results for ANOVA.pdf]

Xinwang Pecan

Xinwang

2024-04-23

R Markdown

This is an R Markdown document. Markdown is a simple formatting syntax for authoring HTML, PDF, and MS Word documents. For more details on using R Markdown see http://rmarkdown.rstudio.com (http://rmarkdown.rstudio.com).

When you click the **Knit** button a document will be generated that includes both content as well as the output of any embedded R code chunks within the document. You can embed an R code chunk like this:

```
# Low pH value data from Holly and Ali
setwd("C:/Users/zhanyou.xu/Box/Xu")
library(openxlsx)

xw= read.xlsx("CRR field data-April 23 2024_ZX.xlsx", sheet = 'Sheet1')
xw= xw[complete.cases(xw),]

head(xw)
```

```
##   number Order Plot Rep Plant Seedstock Provenance Height_cmJ17 Diameter_mmJ17
## 1      1  1036  104   4     6 87MX1-1.2          S           75             19
## 2      2  1251  126   5     1 87MX1-1.2          S           78             15
## 3      3    88    9   1     8 87MX1-1.2          S           25              6
## 4      4  1256  126   5     6 87MX1-1.2          S           32              7
## 5      5  1600  160   7     1 87MX1-1.2          S           33             10
## 6      6  1434  144   6     4 87MX1-1.2          S           41             12
##   BBApr1 TermBO ApWhrlO
## 1      3      1       4
## 2      3      1       3
## 3      4      1       0
## 4      4      1       5
## 5      4      1       4
## 6      4      1       3
```

```
dim(xw)
```

```
## [1] 1156   12
```

```
sum(is.na(xw))
```

```
## [1] 0
```

```r
sum(is.na(xw$Height_cmJ17))
```

```
## [1] 0
```

```r
sum(is.na(xw$Diameter_mmJ17))
```

```
## [1] 0
```

```r
sum(is.na(xw$BBApr1))
```

```
## [1] 0
```

```r
sum(is.na(xw$TermBO))
```

```
## [1] 0
```

```r
sum(is.na(xw$ApWhrlO))
```

```
## [1] 0
```

```r
xw[is.na(xw$Diameter_mmJ17),]
```

```
##  [1] number         Order          Plot           Rep            Plant
##  [6] Seedstock      Provenance     Height_cmJ17   Diameter_mmJ17 BBApr1
## [11] TermBO         ApWhrlO
## <0 rows> (or 0-length row.names)
```

```r
length(unique(xw$Seedstock))
```

```
## [1] 26
```

```r
xw$Seedstock= factor(xw$Seedstock)
xw$Provenance= factor(xw$Provenance)

library(lmerTest)
```

```
## Warning: package 'lmerTest' was built under R version 4.3.3
```

```
## Loading required package: lme4
```

```
## Loading required package: Matrix
```

```
## Warning: package 'Matrix' was built under R version 4.3.3
```

```
##
## Attaching package: 'lmerTest'
```

```
## The following object is masked from 'package:lme4':
##
##     lmer
```

```
## The following object is masked from 'package:stats':
##
##     step
```

```
library(lme4); library(Matrix); library(lmerTest )


# treat Entry or genotypes as random effects to calculate the variance
head(xw)
```

```
##   number Order Plot Rep Plant Seedstock Provenance Height_cmJ17 Diameter_mmJ17
## 1      1  1036  104   4     6 87MX1-1.2          S           75             19
## 2      2  1251  126   5     1 87MX1-1.2          S           78             15
## 3      3    88    9   1     8 87MX1-1.2          S           25              6
## 4      4  1256  126   5     6 87MX1-1.2          S           32              7
## 5      5  1600  160   7     1 87MX1-1.2          S           33             10
## 6      6  1434  144   6     4 87MX1-1.2          S           41             12
##   BBApr1 TermBO ApWhrlO
## 1      3      1       4
## 2      3      1       3
## 3      4      1       0
## 4      4      1       5
## 5      4      1       4
## 6      4      1       3
```

```
m01XW_1= lmer(formula = Height_cmJ17~ 1 + (1|Seedstock) + (1|Rep) , data=xw)
m01XW_2= lmer(formula = Diameter_mmJ17 ~ 1 + (1|Seedstock) + (1|Rep) , data=xw)
m01XW_3= lmer(formula = BBApr1 ~ 1 + (1|Seedstock) + (1|Rep) , data=xw)
m01XW_4= lmer(formula = TermBO~ 1 + (1|Seedstock) + (1|Rep) , data=xw)
```

```
## boundary (singular) fit: see help('isSingular')
```

```
m01XW_5= lmer(formula = ApWhrlO~ 1 + (1|Seedstock)+ (1|Rep) , data=xw)

summary(m01XW_1)
```

```
## Linear mixed model fit by REML. t-tests use Satterthwaite's method [
## lmerModLmerTest]
## Formula: Height_cmJ17 ~ 1 + (1 | Seedstock) + (1 | Rep)
##    Data: xw
##
## REML criterion at convergence: 12934.2
##
## Scaled residuals:
##     Min      1Q  Median      3Q     Max
## -3.5250 -0.6362 -0.1783  0.5463  4.5642
##
## Random effects:
##  Groups    Name        Variance Std.Dev.
##  Seedstock (Intercept) 1769.2   42.06
##  Rep       (Intercept)  700.1   26.46
##  Residual              3905.0   62.49
## Number of obs: 1156, groups:  Seedstock, 26; Rep, 8
##
## Fixed effects:
##             Estimate Std. Error    df t value Pr(>|t|)
## (Intercept)    93.92      12.66 17.57   7.421 8.14e-07 ***
## ---
## Signif. codes:  0 '***' 0.001 '**' 0.01 '*' 0.05 '.' 0.1 ' ' 1
```

```
summary(m01XW_2)
```

```
## Linear mixed model fit by REML. t-tests use Satterthwaite's method [
## lmerModLmerTest]
## Formula: Diameter_mmJ17 ~ 1 + (1 | Seedstock) + (1 | Rep)
##    Data: xw
##
## REML criterion at convergence: 8891.7
##
## Scaled residuals:
##     Min      1Q  Median      3Q     Max
## -2.9115 -0.6608 -0.1592  0.5058  7.3046
##
## Random effects:
##  Groups    Name        Variance Std.Dev.
##  Seedstock (Intercept)  36.54    6.045
##  Rep       (Intercept)  14.58    3.819
##  Residual              119.12   10.914
## Number of obs: 1156, groups:  Seedstock, 26; Rep, 8
##
## Fixed effects:
##             Estimate Std. Error     df t value Pr(>|t|)
## (Intercept)   18.850      1.835 16.871   10.27 1.12e-08 ***
## ---
## Signif. codes:  0 '***' 0.001 '**' 0.01 '*' 0.05 '.' 0.1 ' ' 1
```

```
summary(m01XW_3)
```

```
## Linear mixed model fit by REML. t-tests use Satterthwaite's method [
## lmerModLmerTest]
## Formula: BBApr1 ~ 1 + (1 | Seedstock) + (1 | Rep)
##    Data: xw
##
## REML criterion at convergence: 4028
##
## Scaled residuals:
##     Min      1Q  Median      3Q     Max
## -3.6341 -0.6289 -0.0162  0.7194  2.7301
##
## Random effects:
##  Groups    Name        Variance Std.Dev.
##  Seedstock (Intercept) 0.75078  0.8665
##  Rep       (Intercept) 0.04227  0.2056
##  Residual              1.77001  1.3304
## Number of obs: 1156, groups:  Seedstock, 26; Rep, 8
##
## Fixed effects:
##             Estimate Std. Error     df t value Pr(>|t|)
## (Intercept)    4.381      0.190 28.343   23.06   <2e-16 ***
## ---
## Signif. codes:  0 '***' 0.001 '**' 0.01 '*' 0.05 '.' 0.1 ' ' 1
```

```
summary(m01XW_4)
```

```
## Linear mixed model fit by REML. t-tests use Satterthwaite's method [
## lmerModLmerTest]
## Formula: TermBO ~ 1 + (1 | Seedstock) + (1 | Rep)
##    Data: xw
##
## REML criterion at convergence: 1528.6
##
## Scaled residuals:
##     Min      1Q  Median      3Q     Max
## -1.8538 -0.1414 -0.0731 -0.0230  6.4586
##
## Random effects:
##  Groups    Name        Variance  Std.Dev.
##  Seedstock (Intercept) 3.795e-02 1.948e-01
##  Rep       (Intercept) 2.210e-18 1.486e-09
##  Residual              2.085e-01 4.567e-01
## Number of obs: 1156, groups:  Seedstock, 26; Rep, 8
##
## Fixed effects:
##             Estimate Std. Error       df t value Pr(>|t|)
## (Intercept)  1.09446    0.04067 25.80340   26.91   <2e-16 ***
## ---
## Signif. codes:  0 '***' 0.001 '**' 0.01 '*' 0.05 '.' 0.1 ' ' 1
## optimizer (nloptwrap) convergence code: 0 (OK)
## boundary (singular) fit: see help('isSingular')
```

```
summary(m01XW_5)
```

```
## Linear mixed model fit by REML. t-tests use Satterthwaite's method [
## lmerModLmerTest]
## Formula: ApWhrlO ~ 1 + (1 | Seedstock) + (1 | Rep)
##    Data: xw
##
## REML criterion at convergence: 2607.9
##
## Scaled residuals:
##     Min      1Q  Median      3Q     Max
## -4.5054 -0.2353 -0.0568  0.3959  2.8092
##
## Random effects:
##  Groups    Name        Variance Std.Dev.
##  Seedstock (Intercept) 0.037221 0.1929
##  Rep       (Intercept) 0.001918 0.0438
##  Residual              0.538841 0.7341
## Number of obs: 1156, groups:  Seedstock, 26; Rep, 8
##
## Fixed effects:
##             Estimate Std. Error       df t value Pr(>|t|)
## (Intercept)  3.04402    0.04683 21.54144      65   <2e-16 ***
## ---
## Signif. codes:  0 '***' 0.001 '**' 0.01 '*' 0.05 '.' 0.1 ' ' 1
```

```
vc1=VarCorr(m01XW_1)
print(vc1, comp=c("Variance"))
```

```
##  Groups    Name        Variance
##  Seedstock (Intercept) 1769.2
##  Rep       (Intercept)  700.1
##  Residual              3905.0
```

```
vc1_df=as.data.frame(VarCorr(m01XW_1))
vc2_df=as.data.frame(VarCorr(m01XW_2))
vc3_df=as.data.frame(VarCorr(m01XW_3))
vc4_df=as.data.frame(VarCorr(m01XW_4))
vc5_df=as.data.frame(VarCorr(m01XW_5))




h1= vc1_df[1,4]/(vc1_df[1,4] + vc1_df[3,4]/8)
h2= vc2_df[1,4]/(vc2_df[1,4] + vc2_df[3,4]/8)
h3= vc3_df[1,4]/(vc3_df[1,4] + vc3_df[3,4]/8)
h4= vc4_df[1,4]/(vc4_df[1,4] + vc4_df[3,4]/8)
h5= vc5_df[1,4]/(vc5_df[1,4] + vc5_df[3,4]/8)

print(paste0("The heritabiloity for trait 1 is: ", h1))
```

```
## [1] "The heritabiloity for trait 1 is: 0.783762158468426"
```

```
print(paste0("The heritabiloity for trait 2 is: ", h2))
```

```
## [1] "The heritabiloity for trait 2 is: 0.710469768973313"
```

```
print(paste0("The heritabiloity for trait 3 is: ", h3))
```

```
## [1] "The heritabiloity for trait 3 is: 0.772382197632255"
```

```
print(paste0("The heritabiloity for trait 4 is: ", h4))
```

```
## [1] "The heritabiloity for trait 4 is: 0.592810235434182"
```

```
print(paste0("The heritabiloity for trait 5 is: ", h5))
```

```
## [1] "The heritabiloity for trait 5 is: 0.355921278724788"
```

```
m02Fix_1=  lmer(formula = Height_cmJ17~  Seedstock + (1|Rep), data=xw)
m02Fix_2=  lmer(formula = Diameter_mmJ17~  Seedstock + (1|Rep), data=xw)
m02Fix_3=  lmer(formula = BBApr1~  Seedstock + (1|Rep), data=xw)
m02Fix_4=  lmer(formula = TermBO ~  Seedstock + (1|Rep), data=xw)
```

```
## boundary (singular) fit: see help('isSingular')
```

```
m02Fix_5=  lmer(formula = ApWhrlO~  Seedstock + (1|Rep), data=xw)

summary(m02Fix_1)
```

```
## Linear mixed model fit by REML. t-tests use Satterthwaite's method [
## lmerModLmerTest]
## Formula: Height_cmJ17 ~ Seedstock + (1 | Rep)
##    Data: xw
## 
## REML criterion at convergence: 12671.7
## 
## Scaled residuals: 
##     Min      1Q  Median      3Q     Max 
## -3.6327 -0.6341 -0.1626  0.5491  4.5590 
## 
## Random effects:
##  Groups   Name        Variance Std.Dev.
##  Rep      (Intercept)  703.7   26.53   
##  Residual             3905.0   62.49   
## Number of obs: 1156, groups:  Rep, 8
## 
## Fixed effects:
##                      Estimate Std. Error       df t value Pr(>|t|)    
## (Intercept)           103.341     12.760   20.899   8.099 7.00e-08 ***
## Seedstock87MX4-5.5  157.533     12.164 1123.269  12.951  < 2e-16 ***
## Seedstock87MX5-1.7   10.244     12.843 1123.087   0.798  0.42526    
## Seedstock97CAT11.3  -39.214     12.691 1123.430  -3.090  0.00205 ** 
## SeedstockA-93          6.796     12.117 1123.570   0.561  0.57503    
## SeedstockAllen3      -64.684     15.658 1123.348  -4.131 3.88e-05 ***
## SeedstockAllen4      -31.083     14.177 1123.501  -2.193  0.02855 *  
## SeedstockApache        2.937     11.621 1123.082   0.253  0.80051    
## SeedstockBaker        -5.933     12.244 1123.708  -0.485  0.62808    
## SeedstockBurkett      -2.245     13.144 1123.903  -0.171  0.86441    
## SeedstockChoctaw     -50.206     16.222 1123.955  -3.095  0.00202 ** 
## SeedstockCurtis      -26.761     12.400 1123.018  -2.158  0.03113 *  
## SeedstockElliott     -13.008     12.099 1123.237  -1.075  0.28253    
## SeedstockFrutoso       7.948     12.049 1123.127   0.660  0.50964    
## SeedstockGiles       -20.630     12.543 1123.111  -1.645  0.10031    
## SeedstockIdeal       -36.648     12.797 1123.764  -2.864  0.00426 ** 
## SeedstockMajor       -54.489     13.813 1123.946  -3.945 8.48e-05 ***
## SeedstockMoore       -38.545     13.294 1122.982  -2.899  0.00381 ** 
## SeedstockPeruque     -53.758     16.357 1125.464  -3.287  0.00105 ** 
## SeedstockRiverside     4.792     11.656 1122.890   0.411  0.68108    
## SeedstockSanFelipe    22.898     12.806 1123.706   1.788  0.07404 .  
## SeedstockShoshoni     11.515     12.219 1123.205   0.942  0.34616    
## SeedstockSioux       -13.519     12.941 1123.264  -1.045  0.29643    
## SeedstockStein       -30.338     12.613 1123.227  -2.405  0.01632 *  
## SeedstockVC 1-68      38.412     12.762 1123.016   3.010  0.00267 ** 
## SeedstockWichita     -37.922     12.773 1123.224  -2.969  0.00305 ** 
## ---
## Signif. codes:  0 '***' 0.001 '**' 0.01 '*' 0.05 '.' 0.1 ' ' 1
```

```
##
## Correlation matrix not shown by default, as p = 26 > 12.
## Use print(x, correlation=TRUE)  or
##     vcov(x)        if you need it
```

```
anova(m02Fix_1)
```

```
## Type III Analysis of Variance Table with Satterthwaite's method
##             Sum Sq Mean Sq NumDF  DenDF F value    Pr(>F)
## Seedstock 2103266   84131    25 1123.5  21.544 < 2.2e-16 ***
## ---
## Signif. codes:  0 '***' 0.001 '**' 0.01 '*' 0.05 '.' 0.1 ' ' 1
```

```
summary(m02Fix_2)
```

```
## Linear mixed model fit by REML. t-tests use Satterthwaite's method [
## lmerModLmerTest]
## Formula: Diameter_mmJ17 ~ Seedstock + (1 | Rep)
##    Data: xw
##
## REML criterion at convergence: 8725.7
##
## Scaled residuals:
##     Min      1Q  Median      3Q     Max
## -3.0336 -0.6457 -0.1498  0.5041  7.1832
##
## Random effects:
##  Groups   Name        Variance Std.Dev.
##  Rep      (Intercept)  14.66    3.829
##  Residual             119.13   10.914
## Number of obs: 1156, groups:  Rep, 8
##
## Fixed effects:
##                       Estimate Std. Error         df t value Pr(>|t|)
## (Intercept)           21.23287    2.02863   28.74527  10.467 2.57e-11 ***
## Seedstock87MX4-5.5    20.78932    2.12447 1123.34539   9.786  < 2e-16 ***
## Seedstock87MX5-1.7    -2.78096    2.24312 1123.09130  -1.240 0.215318
## Seedstock97CAT11.3    -7.76436    2.21648 1123.55578  -3.503 0.000478 ***
## SeedstockA-93         -0.43980    2.11624 1123.76518  -0.208 0.835406
## SeedstockAllen3      -10.69442    2.73459 1123.47785  -3.911 9.75e-05 ***
## SeedstockAllen4       -5.80923    2.47592 1123.67521  -2.346 0.019134 *
## SeedstockApache        0.12022    2.02957 1123.08331   0.059 0.952775
## SeedstockBaker        -2.41949    2.13827 1123.96881  -1.132 0.258080
## SeedstockBurkett       0.29672    2.29540 1124.23943   0.129 0.897169
## SeedstockChoctaw      -7.10165    2.83304 1124.33580  -2.507 0.012326 *
## SeedstockCurtis       -4.76003    2.16570 1122.99398  -2.198 0.028159 *
## SeedstockElliott      -1.85690    2.11304 1123.28587  -0.879 0.379708
## SeedstockFrutoso       0.07309    2.10441 1123.14849   0.035 0.972299
## SeedstockGiles        -3.06512    2.19067 1123.12831  -1.399 0.162037
## SeedstockIdeal        -7.34891    2.23482 1124.04354  -3.288 0.001039 **
## SeedstockMajor        -8.42289    2.41224 1124.30971  -3.492 0.000499 ***
## SeedstockMoore        -8.33019    2.32183 1122.93438  -3.588 0.000348 ***
## SeedstockPeruque      -9.30061    2.85614 1126.37503  -3.256 0.001162 **
## SeedstockRiverside    -0.52560    2.03574 1122.81014  -0.258 0.796312
## SeedstockSanFelipe     3.84148    2.23652 1123.97792   1.718 0.086142 .
## SeedstockShoshoni      1.94914    2.13399 1123.25207   0.913 0.361239
## SeedstockSioux        -2.47518    2.26023 1123.34788  -1.095 0.273706
## SeedstockStein        -5.27546    2.20284 1123.28186  -2.395 0.016790 *
## SeedstockVC 1-68       4.12021    2.22893 1122.99159   1.849 0.064791 .
## SeedstockWichita      -7.03821    2.23076 1123.29173  -3.155 0.001647 **
## ---
## Signif. codes:  0 '***' 0.001 '**' 0.01 '*' 0.05 '.' 0.1 ' ' 1
```

```
##
## Correlation matrix not shown by default, as p = 26 > 12.
## Use print(x, correlation=TRUE)  or
##     vcov(x)        if you need it
```

```
anova(m02Fix_2)
```

```
## Type III Analysis of Variance Table with Satterthwaite's method
##           Sum Sq Mean Sq NumDF  DenDF F value    Pr(>F)
## Seedstock  44094  1763.8    25 1123.7  14.806 < 2.2e-16 ***
## ---
## Signif. codes:  0 '***' 0.001 '**' 0.01 '*' 0.05 '.' 0.1 ' ' 1
```

```
summary(m02Fix_3)
```

```
## Linear mixed model fit by REML. t-tests use Satterthwaite's method [
## lmerModLmerTest]
## Formula: BBApr1 ~ Seedstock + (1 | Rep)
##    Data: xw
##
## REML criterion at convergence: 3959.4
##
## Scaled residuals:
##     Min      1Q  Median      3Q     Max
## -3.6808 -0.6323 -0.0399  0.7075  2.8288
##
## Random effects:
##  Groups   Name        Variance Std.Dev.
##  Rep      (Intercept) 0.0438   0.2093
##  Residual             1.7695   1.3302
## Number of obs: 1156, groups:  Rep, 8
##
## Fixed effects:
##                       Estimate Std. Error         df t value Pr(>|t|)
## (Intercept)          5.118e+00  1.981e-01  1.671e+02  25.834  < 2e-16 ***
## Seedstock87MX4-5.5   7.480e-01  2.588e-01  1.125e+03   2.890  0.00393 **
## Seedstock87MX5-1.7  -4.729e-01  2.733e-01  1.124e+03  -1.730  0.08383 .
## Seedstock97CAT11.3  -2.241e+00  2.700e-01  1.125e+03  -8.302 2.91e-16 ***
## SeedstockA-93        3.256e-01  2.577e-01  1.126e+03   1.263  0.20671
## SeedstockAllen3     -1.728e+00  3.331e-01  1.126e+03  -5.189 2.51e-07 ***
## SeedstockAllen4     -1.690e+00  3.016e-01  1.126e+03  -5.605 2.61e-08 ***
## SeedstockApache     -2.463e-01  2.473e-01  1.124e+03  -0.996  0.31937
## SeedstockBaker       9.487e-03  2.604e-01  1.127e+03   0.036  0.97094
## SeedstockBurkett    -7.253e-02  2.795e-01  1.127e+03  -0.260  0.79527
## SeedstockChoctaw    -1.429e+00  3.449e-01  1.128e+03  -4.145 3.66e-05 ***
## SeedstockCurtis     -4.963e-01  2.639e-01  1.124e+03  -1.881  0.06029 .
## SeedstockElliott     5.437e-01  2.574e-01  1.125e+03   2.112  0.03491 *
## SeedstockFrutoso    -5.684e-01  2.564e-01  1.125e+03  -2.217  0.02683 *
## SeedstockGiles      -1.309e+00  2.669e-01  1.125e+03  -4.905 1.07e-06 ***
## SeedstockIdeal      -2.093e+00  2.721e-01  1.127e+03  -7.690 3.19e-14 ***
## SeedstockMajor      -2.586e+00  2.937e-01  1.128e+03  -8.804  < 2e-16 ***
## SeedstockMoore      -6.994e-01  2.829e-01  1.124e+03  -2.472  0.01359 *
## SeedstockPeruque    -2.074e+00  3.471e-01  1.130e+03  -5.974 3.09e-09 ***
## SeedstockRiverside  -5.407e-01  2.481e-01  1.124e+03  -2.180  0.02950 *
## SeedstockSanFelipe  -7.445e-01  2.723e-01  1.127e+03  -2.734  0.00636 **
## SeedstockShoshoni   -4.768e-01  2.600e-01  1.125e+03  -1.834  0.06693 .
## SeedstockSioux      -7.419e-01  2.753e-01  1.125e+03  -2.694  0.00715 **
## SeedstockStein      -4.640e-01  2.684e-01  1.125e+03  -1.729  0.08411 .
## SeedstockVC 1-68     2.304e-01  2.716e-01  1.124e+03   0.848  0.39635
## SeedstockWichita    -6.363e-01  2.718e-01  1.125e+03  -2.341  0.01940 *
## ---
## Signif. codes:  0 '***' 0.001 '**' 0.01 '*' 0.05 '.' 0.1 ' ' 1
```

```
##
## Correlation matrix not shown by default, as p = 26 > 12.
## Use print(x, correlation=TRUE)  or
##     vcov(x)        if you need it
```

```
anova(m02Fix_3)
```

```
## Type III Analysis of Variance Table with Satterthwaite's method
##           Sum Sq Mean Sq NumDF  DenDF F value    Pr(>F)
## Seedstock 805.35  32.214    25 1125.5  18.205 < 2.2e-16 ***
## ---
## Signif. codes:  0 '***' 0.001 '**' 0.01 '*' 0.05 '.' 0.1 ' ' 1
```

```
summary(m02Fix_4)
```

```
## Linear mixed model fit by REML. t-tests use Satterthwaite's method [
## lmerModLmerTest]
## Formula: TermBO ~ Seedstock + (1 | Rep)
##    Data: xw
##
## REML criterion at convergence: 1533.5
##
## Scaled residuals:
##     Min      1Q  Median      3Q     Max
## -2.0242 -0.1340 -0.0547  0.0000  6.4655
##
## Random effects:
##  Groups   Name        Variance  Std.Dev.
##  Rep      (Intercept) 2.497e-19 4.997e-10
##  Residual             2.086e-01 4.567e-01
## Number of obs: 1156, groups:  Rep, 8
##
## Fixed effects:
##                        Estimate Std. Error         df t value Pr(>|t|)
## (Intercept)          1.038e+00  6.274e-02  1.130e+03  16.540  < 2e-16 ***
## Seedstock87MX4-5.5  8.868e-01  8.873e-02  1.130e+03   9.995  < 2e-16 ***
## Seedstock87MX5-1.7  4.971e-01  9.374e-02  1.130e+03   5.303 1.37e-07 ***
## Seedstock97CAT11.3  2.289e-01  9.259e-02  1.130e+03   2.473   0.0136 *
## SeedstockA-93      -3.774e-02  8.831e-02  1.130e+03  -0.427   0.6693
## SeedstockAllen3    -3.774e-02  1.140e-01  1.130e+03  -0.331   0.7408
## SeedstockAllen4     2.678e-02  1.033e-01  1.130e+03   0.259   0.7954
## SeedstockApache     9.139e-03  8.483e-02  1.130e+03   0.108   0.9142
## SeedstockBaker      7.257e-04  8.915e-02  1.130e+03   0.008   0.9935
## SeedstockBurkett   -1.274e-02  9.566e-02  1.130e+03  -0.133   0.8941
## SeedstockChoctaw   -3.774e-02  1.178e-01  1.130e+03  -0.320   0.7487
## SeedstockCurtis     2.349e-02  9.052e-02  1.130e+03   0.259   0.7953
## SeedstockElliott    1.104e-01  8.831e-02  1.130e+03   1.250   0.2115
## SeedstockFrutoso   -3.774e-02  8.792e-02  1.130e+03  -0.429   0.6678
## SeedstockGiles     -3.774e-02  9.151e-02  1.130e+03  -0.412   0.6802
## SeedstockIdeal      7.719e-03  9.315e-02  1.130e+03   0.083   0.9340
## SeedstockMajor     -3.774e-02  1.004e-01  1.130e+03  -0.376   0.7070
## SeedstockMoore      1.490e-02  9.709e-02  1.130e+03   0.153   0.8781
## SeedstockPeruque   -3.774e-02  1.178e-01  1.130e+03  -0.320   0.7487
## SeedstockRiverside -2.186e-02  8.513e-02  1.130e+03  -0.257   0.7974
## SeedstockSanFelipe  3.045e-02  9.315e-02  1.130e+03   0.327   0.7439
## SeedstockShoshoni  -1.851e-02  8.915e-02  1.130e+03  -0.208   0.8356
## SeedstockSioux     -3.774e-02  9.436e-02  1.130e+03  -0.400   0.6893
## SeedstockStein     -3.774e-02  9.204e-02  1.130e+03  -0.410   0.6819
## SeedstockVC 1-68    3.045e-02  9.315e-02  1.130e+03   0.327   0.7439
## SeedstockWichita   -3.774e-02  9.315e-02  1.130e+03  -0.405   0.6855
## ---
## Signif. codes:  0 '***' 0.001 '**' 0.01 '*' 0.05 '.' 0.1 ' ' 1
```

```
##
## Correlation matrix not shown by default, as p = 26 > 12.
## Use print(x, correlation=TRUE)  or
##     vcov(x)        if you need it
```

```
## optimizer (nloptwrap) convergence code: 0 (OK)
## boundary (singular) fit: see help('isSingular')
```

```
anova(m02Fix_4)
```

```
## Type III Analysis of Variance Table with Satterthwaite's method
##           Sum Sq Mean Sq NumDF DenDF F value    Pr(>F)
## Seedstock 51.419  2.0568    25  1130  9.8589 < 2.2e-16 ***
## ---
## Signif. codes:  0 '***' 0.001 '**' 0.01 '*' 0.05 '.' 0.1 ' ' 1
```

```
summary(m02Fix_5)
```

```
## Linear mixed model fit by REML. t-tests use Satterthwaite's method [
## lmerModLmerTest]
## Formula: ApWhrlO ~ Seedstock + (1 | Rep)
##    Data: xw
##
## REML criterion at convergence: 2608.7
##
## Scaled residuals:
##     Min      1Q  Median      3Q     Max
## -4.6685 -0.2917 -0.0574  0.5401  2.8395
##
## Random effects:
##  Groups   Name        Variance Std.Dev.
##  Rep      (Intercept) 0.0020   0.04472
##  Residual             0.5389   0.73410
## Number of obs: 1156, groups:  Rep, 8
##
## Fixed effects:
##                       Estimate Std. Error         df t value Pr(>|t|)
## (Intercept)            2.98256    0.10228  646.70731  29.162  < 2e-16 ***
## Seedstock87MX4-5.5   -0.56591    0.14270 1128.18952  -3.966 7.77e-05 ***
## Seedstock87MX5-1.7   -0.35261    0.15073 1127.17937  -2.339  0.01949 *
## Seedstock97CAT11.3   -0.02777    0.14887 1127.52026  -0.187  0.85204
## SeedstockA-93        -0.12532    0.14205 1128.92521  -0.882  0.37785
## SeedstockAllen3       0.22774    0.18354 1129.80490   1.241  0.21493
## SeedstockAllen4       0.15627    0.16618 1129.58104   0.940  0.34720
## SeedstockApache       0.22103    0.13639 1126.91165   1.621  0.10538
## SeedstockBaker        0.04034    0.14345 1129.72736   0.281  0.77858
## SeedstockBurkett     -0.02889    0.15393 1129.83739  -0.188  0.85118
## SeedstockChoctaw      0.15531    0.18976 1128.22734   0.818  0.41327
## SeedstockCurtis       0.16081    0.14555 1127.06480   1.105  0.26945
## SeedstockElliott      0.01662    0.14199 1126.66428   0.117  0.90685
## SeedstockFrutoso      0.07374    0.14138 1127.86222   0.522  0.60208
## SeedstockGiles        0.23072    0.14718 1127.99872   1.568  0.11725
## SeedstockIdeal        0.31423    0.14991 1129.85768   2.096  0.03629 *
## SeedstockMajor        0.46674    0.16164 1129.35919   2.888  0.00396 **
## SeedstockMoore        0.04392    0.15608 1126.04805   0.281  0.77846
## SeedstockPeruque      0.30912    0.19028 1098.49687   1.625  0.10455
## SeedstockRiverside    0.08373    0.13686 1126.04366   0.612  0.54079
## SeedstockSanFelipe    0.09247    0.14997 1129.96134   0.617  0.53762
## SeedstockShoshoni     0.17131    0.14337 1127.70799   1.195  0.23236
## SeedstockSioux       -0.09305    0.15178 1128.84844  -0.613  0.53997
## SeedstockStein        0.28045    0.14800 1127.36883   1.895  0.05835 .
## SeedstockVC 1-68     -0.27846    0.14979 1127.29142  -1.859  0.06329 .
## SeedstockWichita      0.17838    0.14983 1128.47535   1.191  0.23407
## ---
## Signif. codes:  0 '***' 0.001 '**' 0.01 '*' 0.05 '.' 0.1 ' ' 1
```

```
##
## Correlation matrix not shown by default, as p = 26 > 12.
## Use print(x, correlation=TRUE)  or
##     vcov(x)        if you need it
```

```
anova(m02Fix_5)
```

```
## Type III Analysis of Variance Table with Satterthwaite's method
##           Sum Sq Mean Sq NumDF  DenDF F value    Pr(>F)
## Seedstock  54.82  2.1928    25 1123.9   4.069 1.179e-10 ***
## ---
## Signif. codes:  0 '***' 0.001 '**' 0.01 '*' 0.05 '.' 0.1 ' ' 1
```

```
df1= data.frame(anova(m02Fix_1))
df2= data.frame(anova(m02Fix_2))
df3= data.frame(anova(m02Fix_3))
df4= data.frame(anova(m02Fix_4))
df5= data.frame(anova(m02Fix_5))

df_all= rbind(df1, df2, df3, df4, df5)
df_all
```

```
##                  Sum.Sq      Mean.Sq NumDF    DenDF   F.value       Pr..F.
## Seedstock  2.103266e+06 84130.627918    25 1123.535 21.544322 2.416684e-78
## Seedstock1 4.409367e+04  1763.746679    25 1123.709 14.805789 1.328348e-53
## Seedstock2 8.053480e+02    32.213918    25 1125.494 18.204708 2.273551e-66
## Seedstock3 5.141879e+01     2.056752    25 1130.000  9.858888 4.022356e-34
## Seedstock4 5.482038e+01     2.192815    25 1123.925  4.069010 1.179222e-10
```

```
write.csv(df_all, "ANOVA_Table_2.csv", row.names = F)

library(ggplot2)
library(ggpubr)
library(tidyverse)
```

```
## ── Attaching core tidyverse packages ──────────────────────── tidyverse 2.0.0 ──
## ✔ dplyr     1.1.4     ✔ readr     2.1.5
## ✔ forcats   1.0.0     ✔ stringr   1.5.1
## ✔ lubridate 1.9.3     ✔ tibble    3.2.1
## ✔ purrr     1.0.2     ✔ tidyr     1.3.1
```

```
## ── Conflicts ──────────────────────────────────────────── tidyverse_conflicts() ──
## ✖ tidyr::expand() masks Matrix::expand()
## ✖ dplyr::filter() masks stats::filter()
## ✖ dplyr::lag()    masks stats::lag()
## ✖ tidyr::pack()   masks Matrix::pack()
## ✖ tidyr::unpack() masks Matrix::unpack()
## ℹ Use the conflicted package (<http://conflicted.r-lib.org/>) to force all conflicts to becom
e errors
```

```
library(broom)
#library(AICcmodavg)
head(xw)
```

```
##   number Order Plot Rep Plant Seedstock Provenance Height_cmJ17 Diameter_mmJ17
## 1      1  1036  104   4     6 87MX1-1.2          S           75             19
## 2      2  1251  126   5     1 87MX1-1.2          S           78             15
## 3      3    88    9   1     8 87MX1-1.2          S           25              6
## 4      4  1256  126   5     6 87MX1-1.2          S           32              7
## 5      5  1600  160   7     1 87MX1-1.2          S           33             10
## 6      6  1434  144   6     4 87MX1-1.2          S           41             12
##   BBApr1 TermBO ApWhrlO
## 1      3      1       4
## 2      3      1       3
## 3      4      1       0
## 4      4      1       5
## 5      4      1       4
## 6      4      1       3
```

```
names(xw)
```

```
##  [1] "number"         "Order"          "Plot"           "Rep"
##  [5] "Plant"          "Seedstock"      "Provenance"     "Height_cmJ17"
##  [9] "Diameter_mmJ17" "BBApr1"         "TermBO"         "ApWhrlO"
```

```
library(car)
```

```
## Loading required package: carData
##
## Attaching package: 'car'
##
## The following object is masked from 'package:dplyr':
##
##     recode
##
## The following object is masked from 'package:purrr':
##
##     some
```

```
table(xw$Seedstock, xw$Rep)
```

```
## 
##               1  2  3  4  5  6  7  8
##   87MX1-1.2 10  9 10  5  9  6  1  3
##   87MX4-5.5  9 10  7 10 10  3  3  1
##   87MX5-1.7  7  9  5  3  7  5  4  3
##   97CAT11.3  8 10  8  4  8  3  4  0
##   A-93      10  8  6  4  9 10  6  1
##   Allen3     6  6  8  0  2  0  0  1
##   Allen4     8  4  2  7  7  2  1  0
##   Apache    10 10 10  7  9  8  6  4
##   Baker     10  9  4  8  9  3  7  2
##   Burkett    8  5  4  3  8  3  7  2
##   Choctaw    5  0  9  0  7  0  0  0
##   Curtis     9 10  7  7  8  2  3  3
##   Elliott    9 10 10  3  7  6  3  6
##   Frutoso    8  7  9  9  7 10  3  2
##   Giles      5  7  7  8  7  8  3  2
##   Ideal     10  1 10  3  9  4  6  1
##   Major      6 10  0  9  5  4  0  0
##   Moore      7  7  7  4  8  2  2  1
##   Peruque    0  4  0  9  0  8  0  0
##   Riverside 10 10  9  7 10 10  3  4
##   SanFelipe  4  8  2  9  9 10  1  1
##   Shoshoni  10  7 10 10  7  4  3  1
##   Sioux     10  5  3  3 10  4  4  3
##   Stein      9  9  6  6  8  3  4  1
##   VC 1-68    8  3 10  6  8  4  3  2
##   Wichita    9  8  6  6 10  0  3  2
```

```
table(xw$Provenance, xw$Rep)
```

```
## 
##      1  2  3  4  5  6  7  8
##   E 53 54 42 30 49 26 25 13
##   M 52 33 48 32 51 20 19 12
##   N 11 21  7 26 12 20  3  2
##   S 34 35 31 27 33 24 11  9
##   W 55 43 41 35 53 32 22 10
```

```
xw$Rep= factor(xw$Rep)
aov1=aov(Height_cmJ17~ Seedstock *Rep   , data = xw )
aov2=aov(Diameter_mmJ17~ Seedstock*Rep   , data = xw )
aov3=aov(BBApr1~  Seedstock*Rep   , data = xw )
aov4=aov(TermBO~ Seedstock*Rep  , data = xw )
aov5=aov(ApWhrlO~ Seedstock*Rep  , data = xw )

#Anova(aov3, type="III")

summary(aov1)
```

```
##                 Df  Sum Sq Mean Sq F value   Pr(>F)
## Seedstock      25 2120678   84827  25.812  < 2e-16 ***
## Rep             7  551714   78816  23.983  < 2e-16 ***
## Seedstock:Rep 156 1206921    7737   2.354 3.91e-15 ***
## Residuals     967 3177885    3286
## ---
## Signif. codes:  0 '***' 0.001 '**' 0.01 '*' 0.05 '.' 0.1 ' ' 1
```

```
summary(aov2)
```

```
##                 Df Sum Sq Mean Sq F value   Pr(>F)
## Seedstock      25  44793  1791.7  16.984  < 2e-16 ***
## Rep             7  11883  1697.6  16.091  < 2e-16 ***
## Seedstock:Rep 156  31738   203.4   1.928 2.64e-09 ***
## Residuals     967 102015   105.5
## ---
## Signif. codes:  0 '***' 0.001 '**' 0.01 '*' 0.05 '.' 0.1 ' ' 1
```

```
summary(aov3)
```

```
##                 Df Sum Sq Mean Sq F value   Pr(>F)
## Seedstock      25  799.4   31.97  19.360  < 2e-16 ***
## Rep             7   50.0    7.15   4.329 9.86e-05 ***
## Seedstock:Rep 156  389.9    2.50   1.513 0.000163 ***
## Residuals     967 1597.1    1.65
## ---
## Signif. codes:  0 '***' 0.001 '**' 0.01 '*' 0.05 '.' 0.1 ' ' 1
```

```
summary(aov4)
```

```
##                 Df Sum Sq Mean Sq F value Pr(>F)    
## Seedstock      25  51.42  2.0568   9.380 <2e-16 ***
## Rep             7   1.03  0.1474   0.672  0.696    
## Seedstock:Rep 156  22.66  0.1453   0.663  0.999    
## Residuals     967 212.04  0.2193                   
## ---
## Signif. codes:  0 '***' 0.001 '**' 0.01 '*' 0.05 '.' 0.1 ' ' 1
```

```
summary(aov5)
```

```
##                 Df Sum Sq Mean Sq F value   Pr(>F)    
## Seedstock      25   54.8  2.1938   4.236 3.26e-11 ***
## Rep             7    5.3  0.7597   1.467   0.1753    
## Seedstock:Rep 156  104.9  0.6724   1.298   0.0127 *  
## Residuals     967  500.8  0.5178                     
## ---
## Signif. codes:  0 '***' 0.001 '**' 0.01 '*' 0.05 '.' 0.1 ' ' 1
```

```
library(agricolae)
```

```
## Warning: package 'agricolae' was built under R version 4.3.3
```

```
tukey.test_1 <- TukeyHSD(aov1)

plot(tukey.test_1)
```

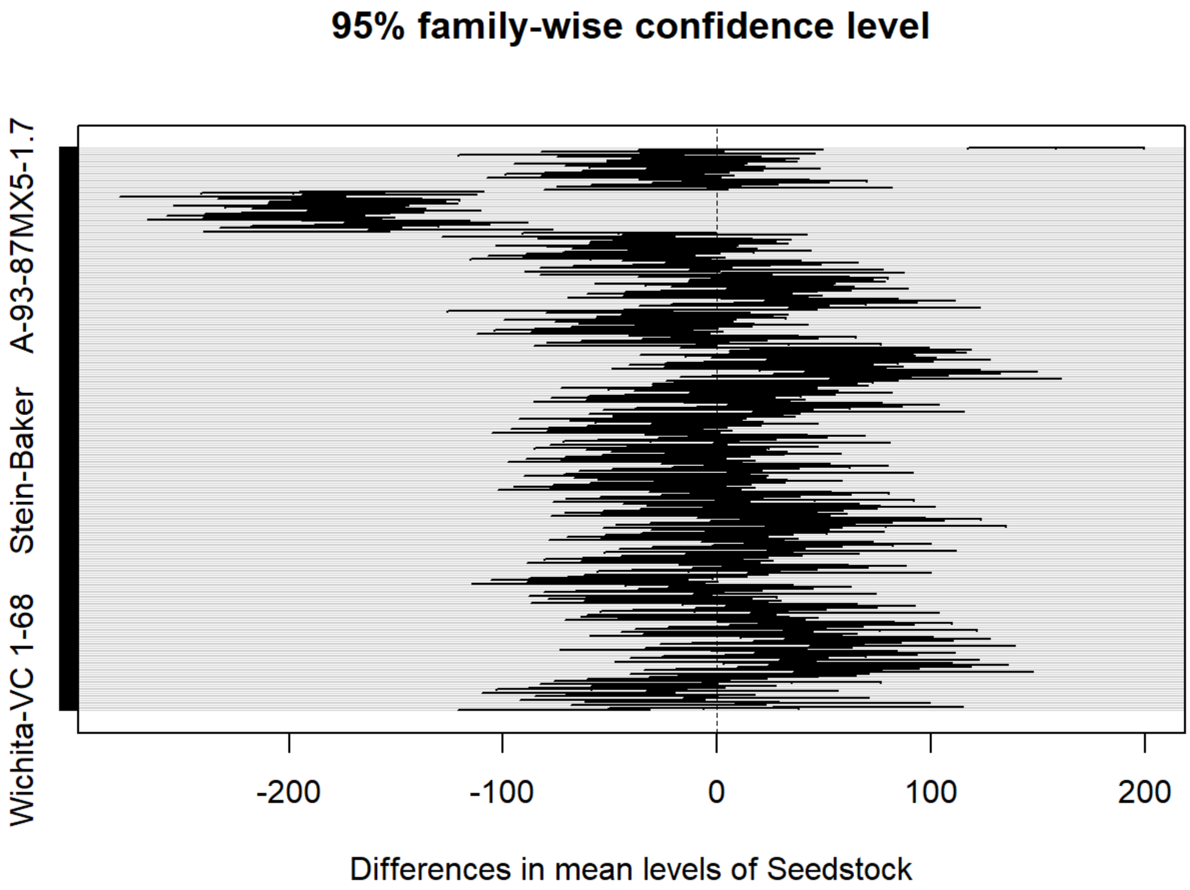

95% family-wise confidence level
A-93-87MX5-1.7
Stein-Baker
Wichita-VC 1-68
-200
-100
0
100
200
Differences in mean levels of Seedstock

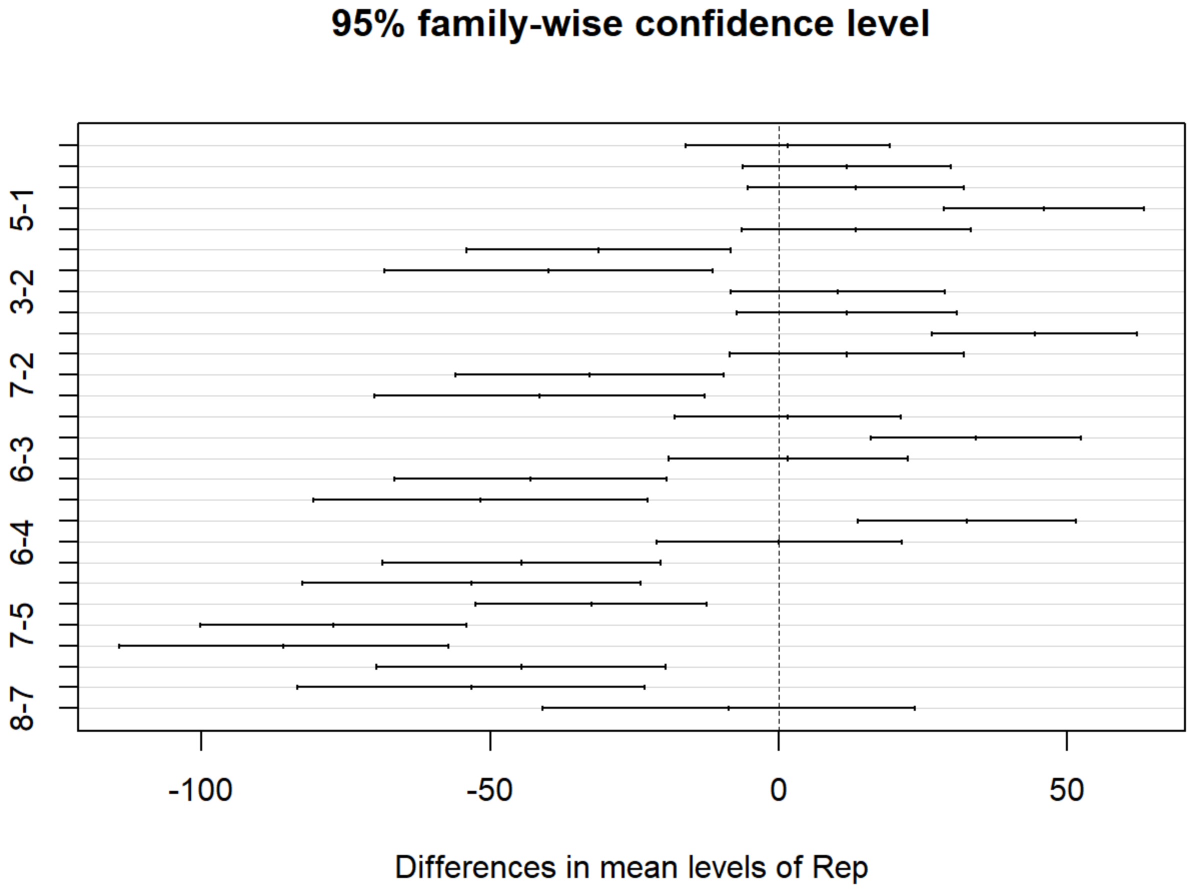

95% family-wise confidence level
5-1
3-2
7-2
6-3
6-4
7-5
8-7
-100
-50
0
50
Differences in mean levels of Rep

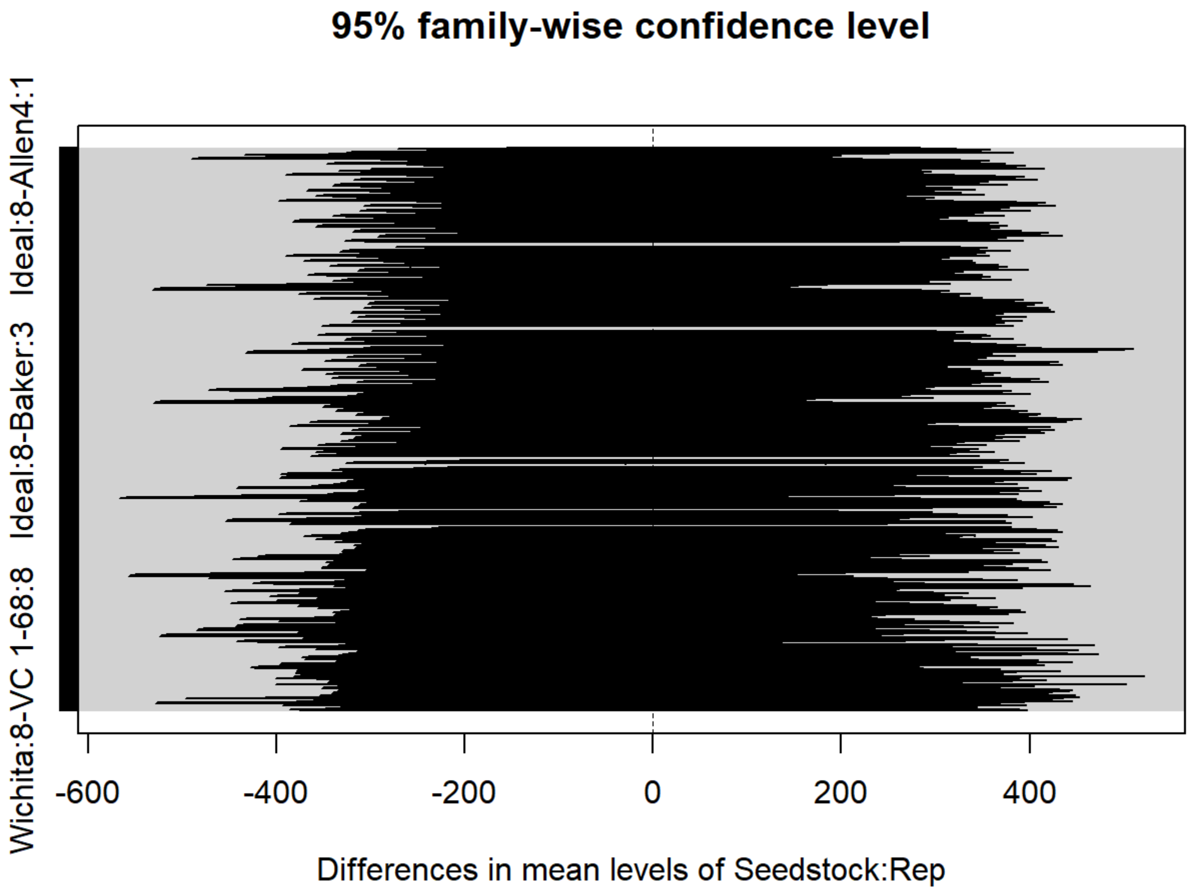


```
tukey.test_1 <- HSD.test(aov1, trt = 'Seedstock')



tukey.test_2 <- TukeyHSD(aov2)

plot(tukey.test_2)
```

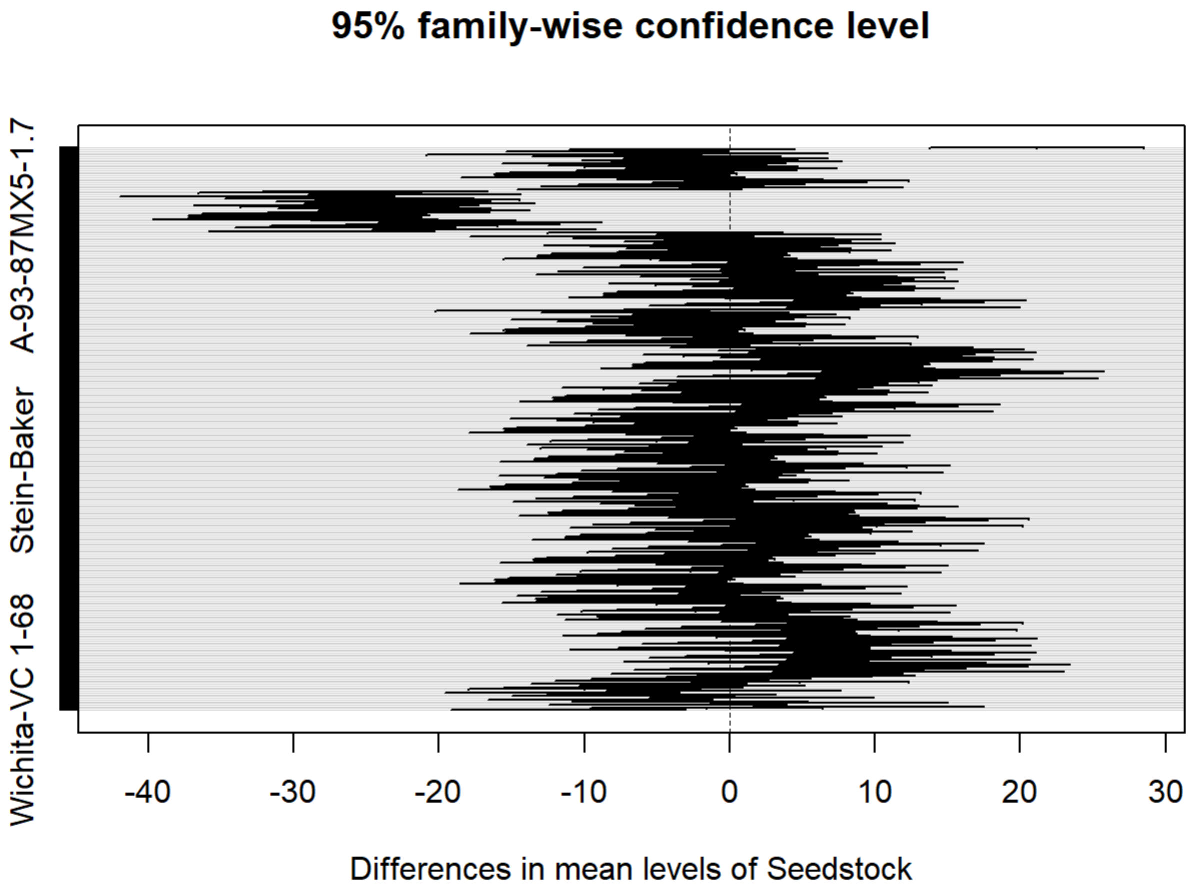

95% family-wise confidence level
Wichita-VC 1-68
Stein-Baker
A-93-87MX5-1.7
-40
-30
-20
-10
0
10
20
30
Differences in mean levels of Seedstock

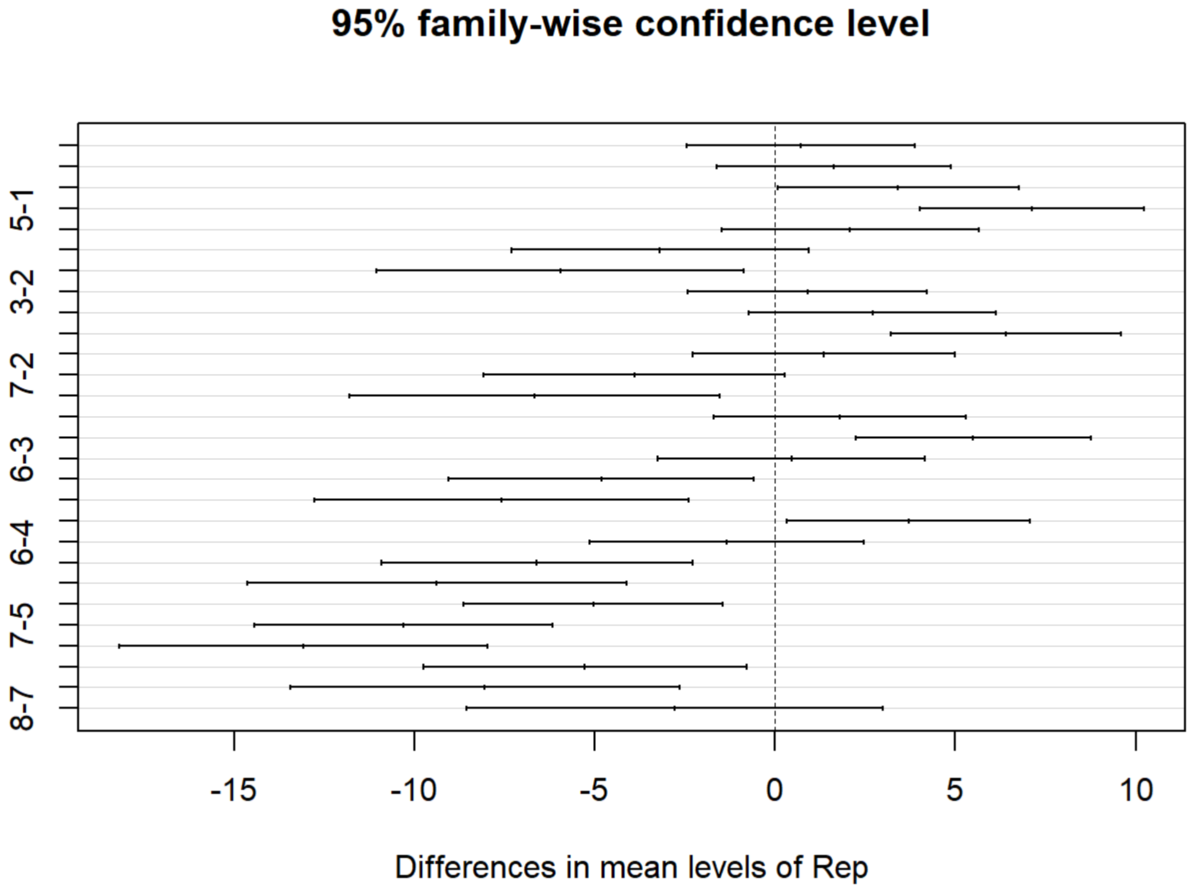

95% family-wise confidence level
5-1
3-2
7-2
6-3
6-4
7-5
8-7
-15
-10
-5
0
5
10
Differences in mean levels of Rep

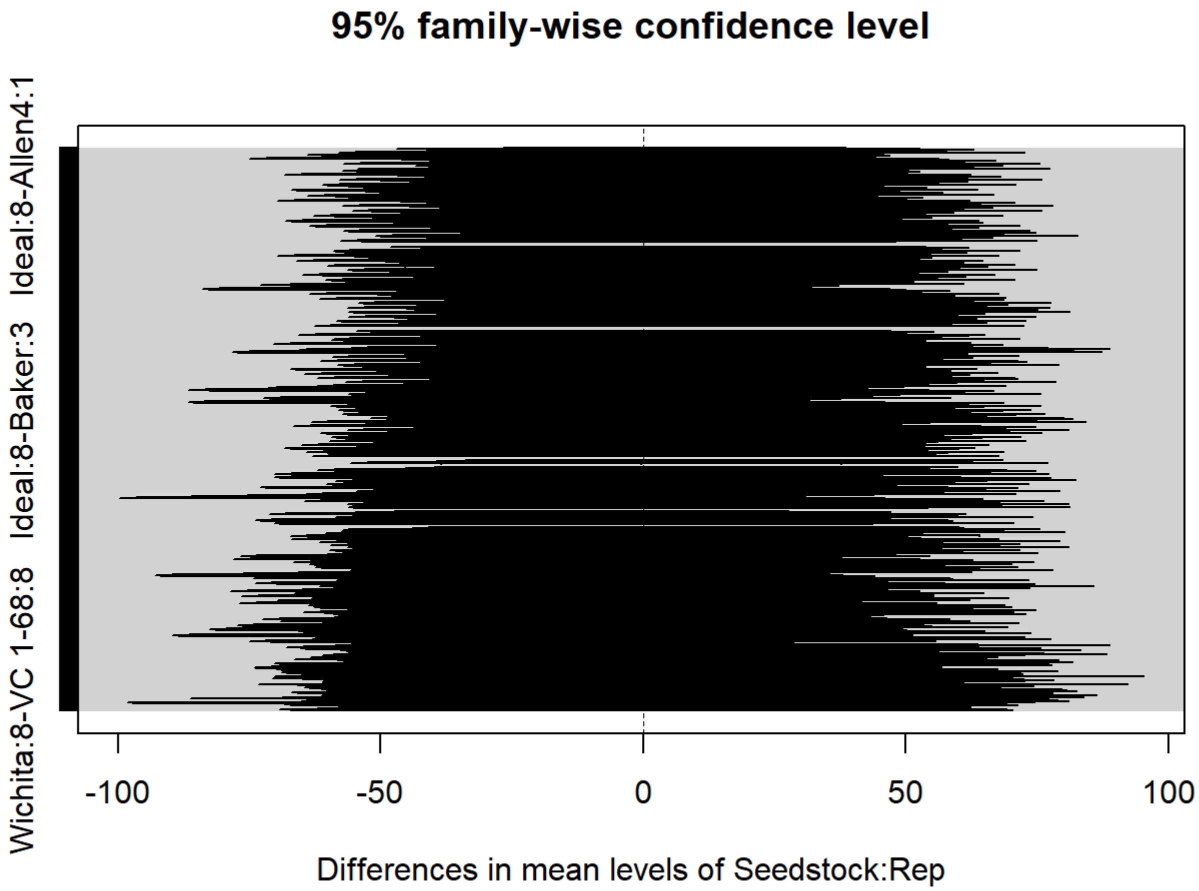


```
tukey.test_2 <- HSD.test(aov2, trt = 'Seedstock')
tukey.test_2
```

```
## $statistics
##    MSerror  Df     Mean       CV
##   105.4966 967 20.53979 50.00612
##
## $parameters
##    test    name.t ntr StudentizedRange alpha
##   Tukey Seedstock  26         5.216432  0.05
##
## $means
##            Diameter_mmJ17       std  r       se Min Max   Q25  Q50   Q75
## 87MX1-1.2       22.20755 14.567295 53 1.410851   6  92 14.00 17.0 30.00
## 87MX4-5.5       43.33962 21.909730 53 1.410851   9 123 31.00 41.0 50.00
## 87MX5-1.7       18.93023 11.498230 43 1.566336   5  50 10.00 15.0 26.00
## 97CAT11.3       14.48889  6.837803 45 1.531133   5  31  9.00 13.0 20.00
## A-93            21.61111 12.166171 54 1.397727   5  50 11.25 18.0 28.75
## Allen3          10.82609  4.355270 23 2.141684   5  20  7.50 10.0 13.00
## Allen4          17.16129  9.637070 31 1.844754   5  43  9.50 15.0 22.50
## Apache          21.89062 11.309663 64 1.283894   6  55 12.00 19.5 26.25
## Baker           19.48077 10.856444 52 1.424353   5  60 12.00 17.5 23.25
## Burkett         21.97500 13.434528 40 1.624012   6  70 11.50 19.5 29.00
## Choctaw         16.28571  7.281091 21 2.241349   5  36 11.00 15.0 20.00
## Curtis          17.22449  8.586000 49 1.467308   6  41 11.00 15.0 20.00
## Elliott         19.55556  9.394231 54 1.397727   5  49 11.50 19.0 23.75
## Frutoso         22.29091 12.727446 55 1.384962   7  50 13.00 17.0 31.50
## Giles           19.23404  8.837206 47 1.498202   5  42 13.00 18.0 25.50
## Ideal           14.75000  7.906797 44 1.548435   7  44  9.00 11.5 18.00
## Major           14.35294  6.531427 34 1.761488   4  29 11.00 14.0 17.75
## Moore           14.13158  7.534322 38 1.666201   4  35  9.25 12.0 18.75
## Peruque         13.47619  8.164674 21 2.241349   5  41  8.00 12.0 15.00
## Riverside       21.55556 12.066720 63 1.294044   7  77 12.50 18.0 27.00
## SanFelipe       26.84091 12.929043 44 1.548435   8  64 17.75 26.0 34.25
## Shoshoni        24.23077 10.634047 52 1.424353   8  44 14.00 24.5 33.00
## Sioux           19.57143 11.850991 42 1.584873   7  58 11.00 17.0 22.75
## Stein           16.89130  8.028362 46 1.514399   6  40 10.25 15.0 23.75
## VC 1-68         26.40909 12.479412 44 1.548435   8  57 17.00 23.0 35.00
## Wichita         15.31818  8.878437 44 1.548435   5  50 10.75 13.0 17.25
##
## $comparison
## NULL
##
## $groups
##            Diameter_mmJ17 groups
## 87MX4-5.5       43.33962      a
## SanFelipe       26.84091      b
## VC 1-68         26.40909      b
## Shoshoni        24.23077     bc
## Frutoso         22.29091    bcd
## 87MX1-1.2       22.20755    bcd
## Burkett         21.97500   bcde
## Apache          21.89062   bcde
## A-93            21.61111   bcde
## Riverside       21.55556   bcde
```

```
## Sioux           19.57143  bcdef
## Elliott         19.55556  bcdef
## Baker           19.48077  bcdef
## Giles           19.23404  bcdef
## 87MX5-1.7       18.93023  bcdef
## Curtis          17.22449   cdef
## Allen4          17.16129   cdef
## Stein           16.89130   cdef
## Choctaw         16.28571   cdef
## Wichita         15.31818    def
## Ideal           14.75000    def
## 97CAT11.3       14.48889     ef
## Major           14.35294     ef
## Moore           14.13158     ef
## Peruque         13.47619     ef
## Allen3          10.82609      f
##
## attr(,"class")
## [1] "group"
```

```
tukey.test_3 <- TukeyHSD(aov3)

plot(tukey.test_3)
```

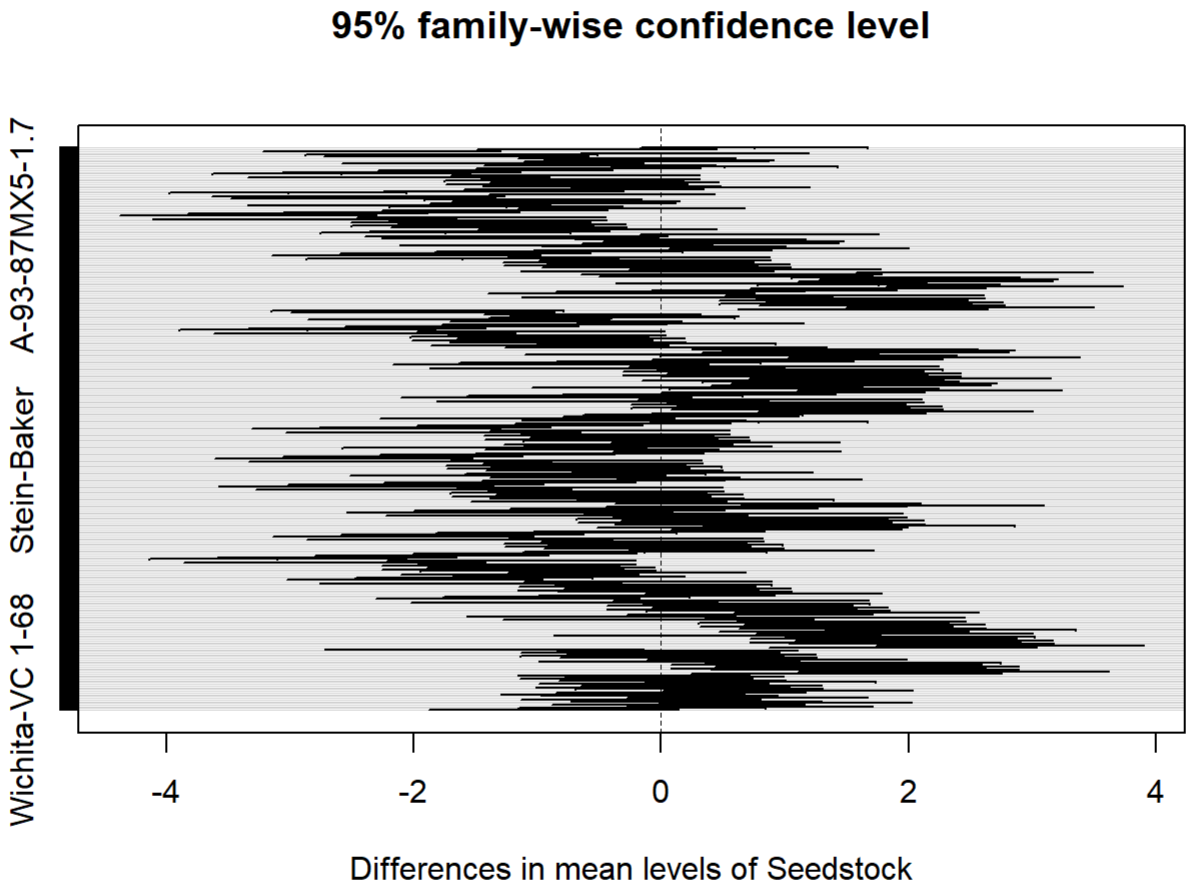

95% family-wise confidence level
A-93-87MX5-1.7
Stein-Baker
Wichita-VC 1-68
-4
-2
0
2
4
Differences in mean levels of Seedstock

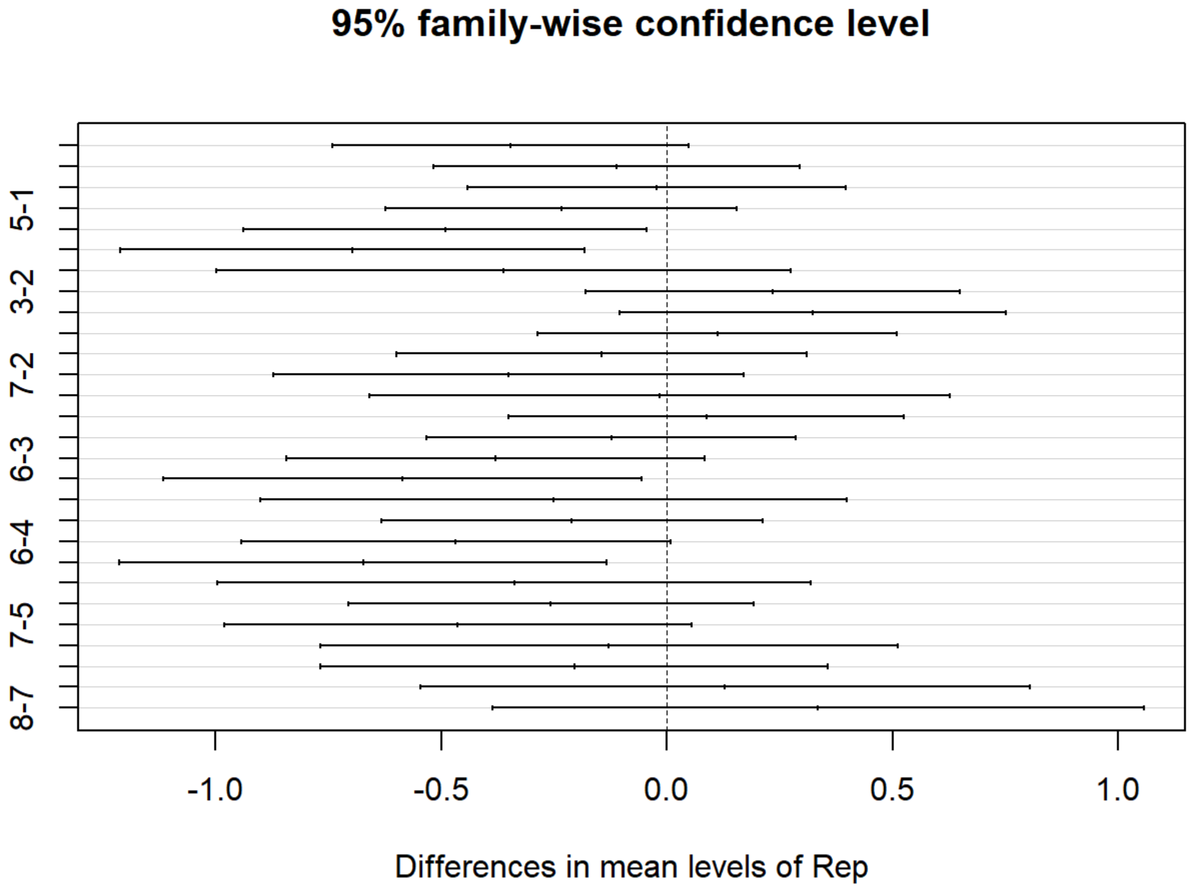

95% family-wise confidence level
5-1
3-2
7-2
6-3
6-4
7-5
8-7
-1.0
-0.5
0.0
0.5
1.0
Differences in mean levels of Rep

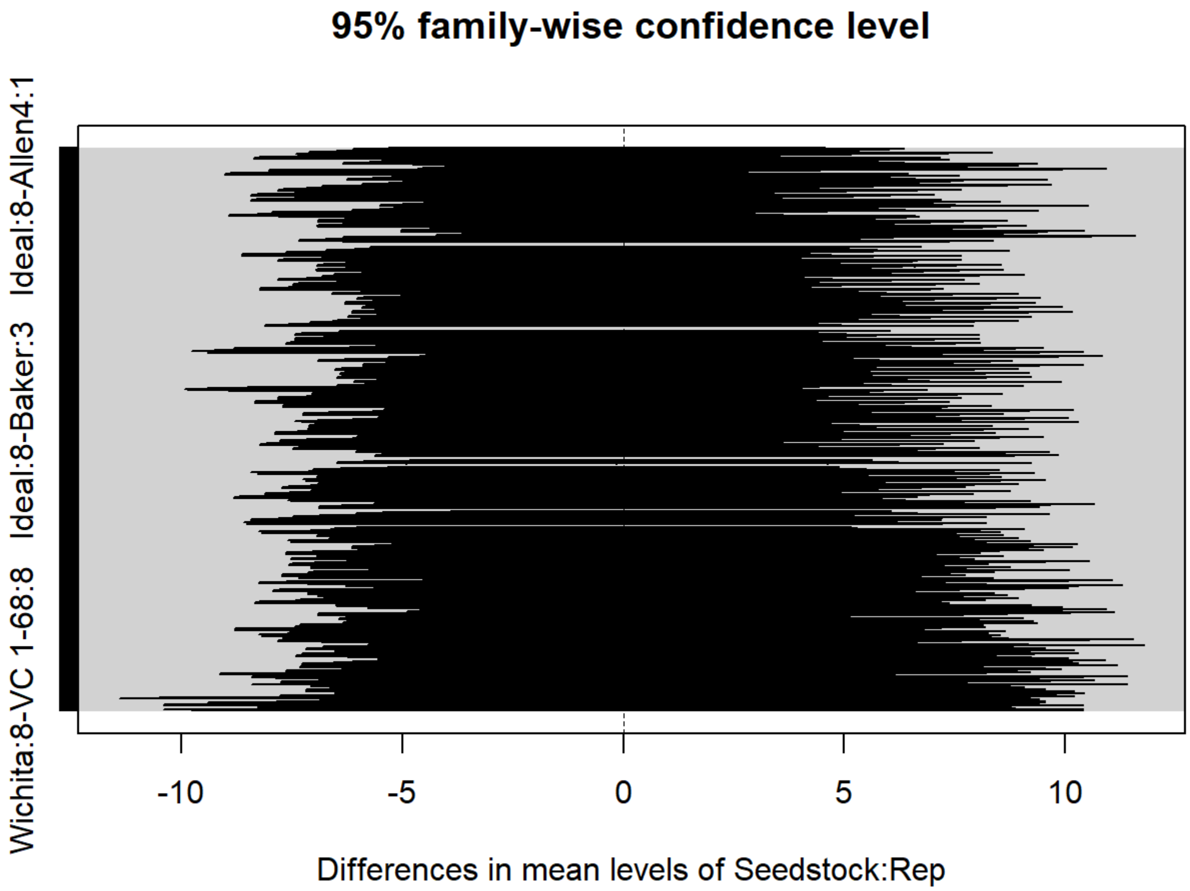


```
tukey.test_3 <- HSD.test(aov3, trt = 'Seedstock')
tukey.test_3
```

```
## $statistics
##    MSerror  Df     Mean       CV
##   1.651576 967 4.547578 28.25981
##
## $parameters
##    test    name.t ntr StudentizedRange alpha
##   Tukey Seedstock  26         5.216432  0.05
##
## $means
##             BBApr1      std  r        se Min Max  Q25 Q50  Q75
## 87MX1-1.2 5.169811 1.266975 53 0.1765271   3   8 4.00 5.0 6.00
## 87MX4-5.5 5.924528 1.491631 53 0.1765271   1   8 5.00 6.0 7.00
## 87MX5-1.7 4.651163 1.088549 43 0.1959815   2   8 4.00 4.0 5.00
## 97CAT11.3 2.911111 1.124565 45 0.1915769   1   5 2.00 3.0 4.00
## A-93      5.444444 1.513046 54 0.1748849   2   8 4.00 6.0 6.00
## Allen3    3.478261 1.274561 23 0.2679695   1   6 3.00 3.0 4.00
## Allen4    3.516129 1.028623 31 0.2308174   1   6 3.00 3.0 4.00
## Apache    4.890625 1.470527 64 0.1606421   2   8 4.00 5.0 6.00
## Baker     5.153846 1.319308 52 0.1782164   2   8 4.00 5.0 6.00
## Burkett   5.050000 1.501282 40 0.2031979   2   8 4.00 5.0 6.00
## Choctaw   3.809524 1.289149 21 0.2804398   1   6 3.00 4.0 4.00
## Curtis    4.673469 1.197006 49 0.1835910   1   7 4.00 5.0 6.00
## Elliott   5.685185 1.225885 54 0.1748849   4   8 5.00 6.0 7.00
## Frutoso   4.581818 1.396966 55 0.1732878   1   7 4.00 5.0 5.50
## Giles     3.829787 1.403709 47 0.1874564   1   7 3.00 4.0 5.00
## Ideal     3.068182 1.227547 44 0.1937416   1   6 2.00 3.0 4.00
## Major     2.588235 1.131308 34 0.2203991   1   5 2.00 2.0 3.75
## Moore     4.473684 1.502014 38 0.2084767   1   7 4.00 4.0 5.75
## Peruque   3.047619 1.465476 21 0.2804398   1   7 2.00 3.0 4.00
## Riverside 4.603175 1.326403 63 0.1619120   2   8 4.00 4.0 6.00
## SanFelipe 4.386364 1.528275 44 0.1937416   1   7 3.75 4.0 5.00
## Shoshoni  4.711538 1.499497 52 0.1782164   1   7 4.00 5.0 6.00
## Sioux     4.404762 1.482574 42 0.1983009   1   7 3.25 4.5 5.75
## Stein     4.695652 1.008179 46 0.1894831   3   7 4.00 4.0 5.00
## VC 1-68   5.409091 1.451834 44 0.1937416   3   8 4.00 5.0 6.25
## Wichita   4.545455 1.265914 44 0.1937416   2   8 4.00 4.0 5.00
##
## $comparison
## NULL
##
## $groups
##             BBApr1 groups
## 87MX4-5.5 5.924528      a
## Elliott   5.685185     ab
## A-93      5.444444    abc
## VC 1-68   5.409091   abcd
## 87MX1-1.2 5.169811  abcde
## Baker     5.153846  abcde
## Burkett   5.050000  abcde
## Apache    4.890625   bcde
## Shoshoni  4.711538   cdef
## Stein     4.695652   cdef
```

```
## Curtis    4.673469   cdef
## 87MX5-1.7 4.651163   cdef
## Riverside 4.603175   cdef
## Frutoso   4.581818   cdef
## Wichita   4.545455  cdefg
## Moore     4.473684  cdefg
## Sioux     4.404762   defg
## SanFelipe 4.386364    efg
## Giles     3.829787    fgh
## Choctaw   3.809524   fghi
## Allen4    3.516129    ghi
## Allen3    3.478261    ghi
## Ideal     3.068182     hi
## Peruque   3.047619     hi
## 97CAT11.3 2.911111     hi
## Major     2.588235      i
##
## attr(,"class")
## [1] "group"
```

```
tukey.test_4 <- TukeyHSD(aov4)

plot(tukey.test_4)
```

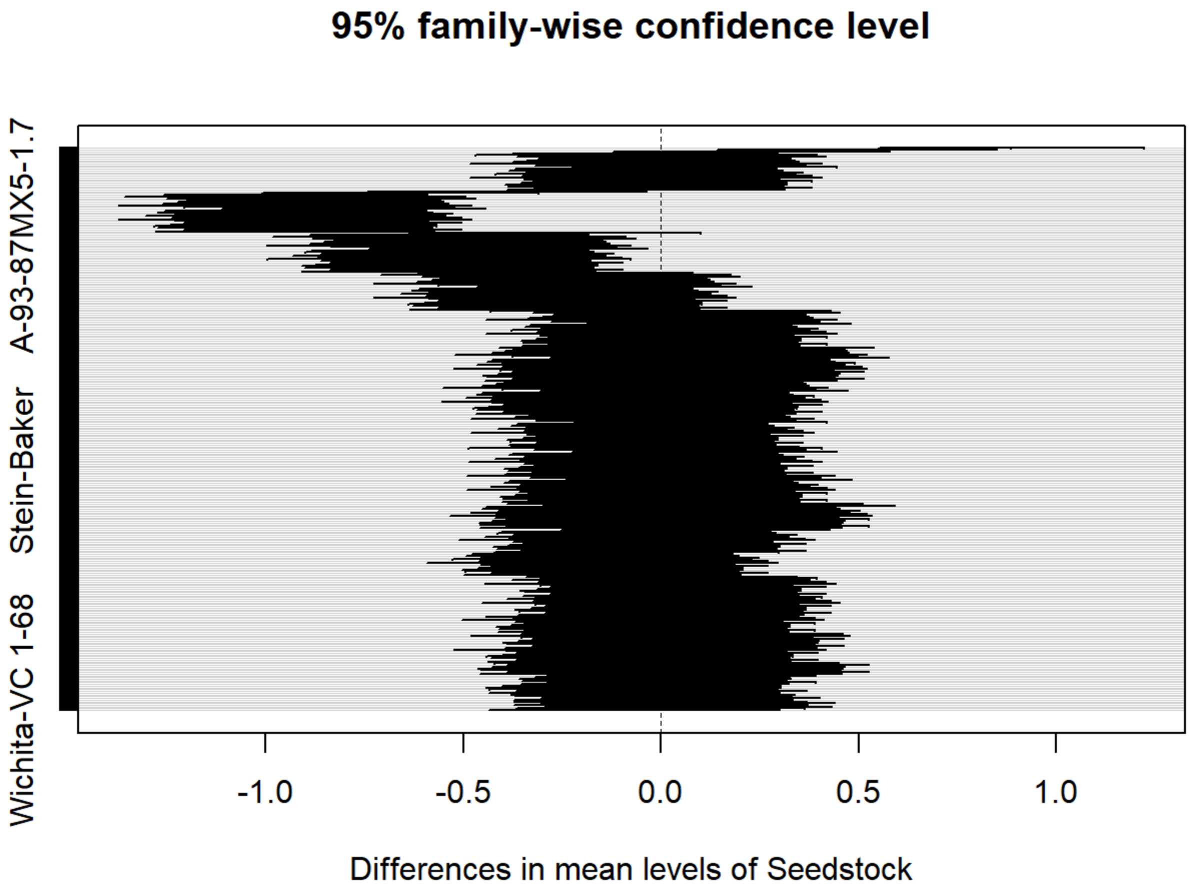
95% family-wise confidence level
A-93-87MX5-1.7
Stein-Baker
Wichita-VC 1-68
-1.0
-0.5
0.0
0.5
1.0
Differences in mean levels of Seedstock

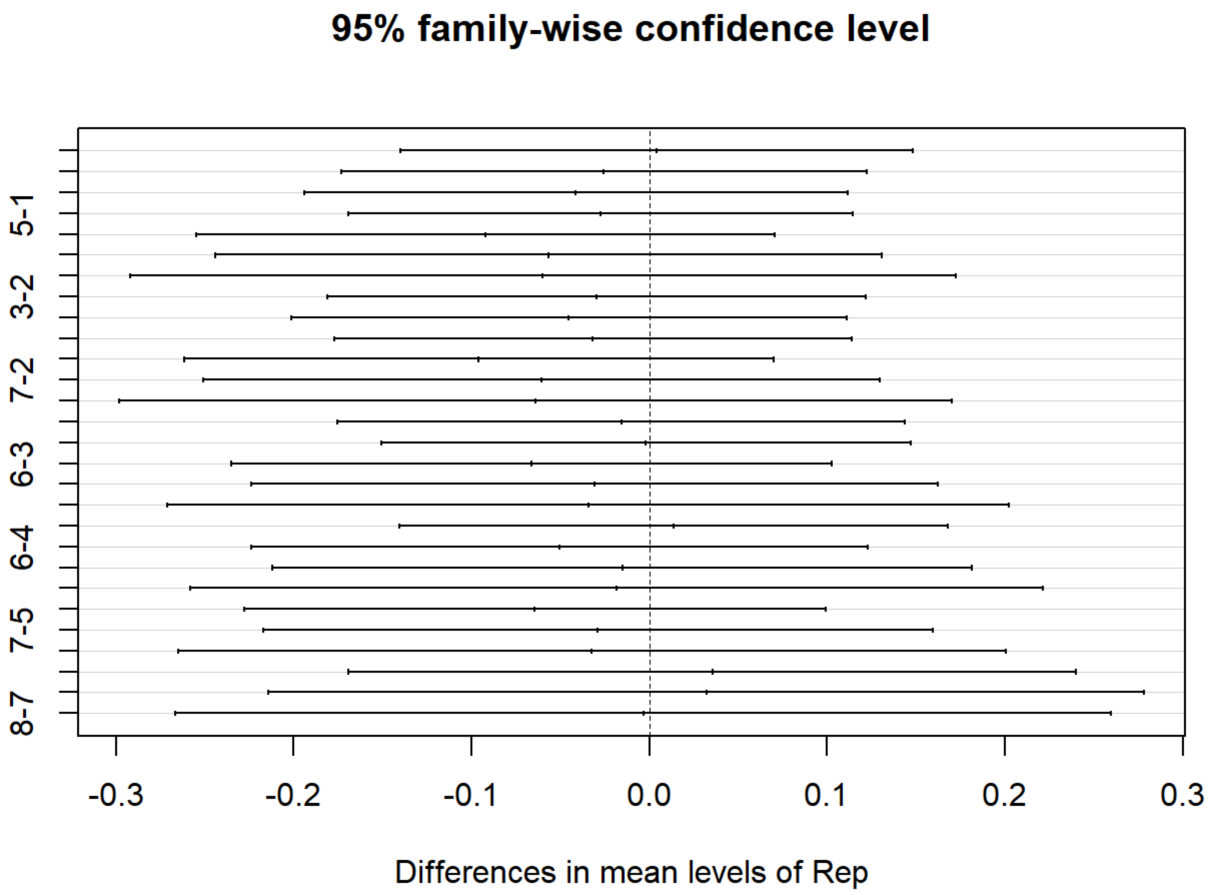
95% family-wise confidence level
5-1
3-2
7-2
6-3
6-4
7-5
8-7
-0.3
-0.2
-0.1
0.0
0.1
0.2
0.3
Differences in mean levels of Rep

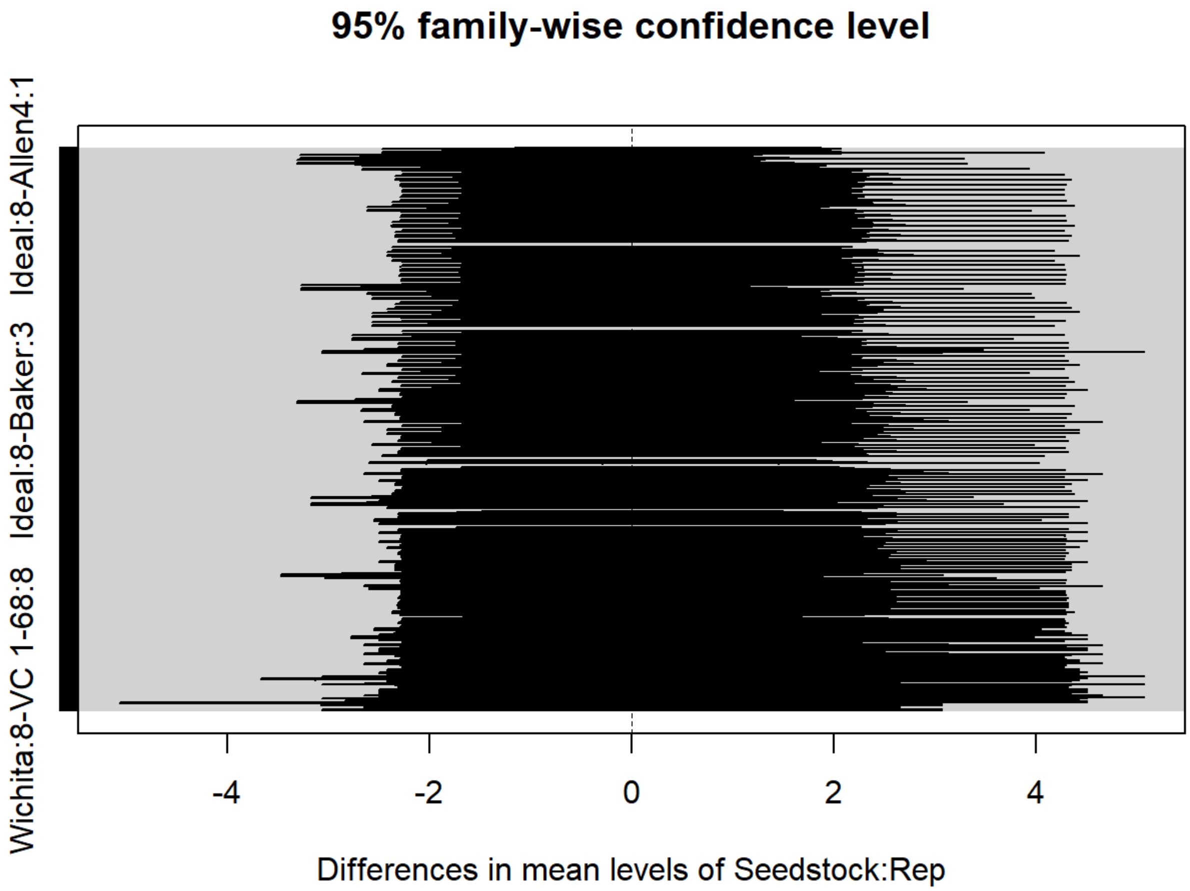


```
tukey.test_4 <- HSD.test(aov4, trt = 'Seedstock')
tukey.test_4
```

```
## $statistics
##     MSerror  Df     Mean       CV
##   0.2192803 967 1.101211 42.52352
##
## $parameters
##    test    name.t ntr StudentizedRange alpha
##   Tukey Seedstock  26         5.216432  0.05
##
## $means
##              TermBO       std  r         se Min Max Q25 Q50 Q75
## 87MX1-1.2 1.037736 0.1923802 53 0.06432235   1   2   1   1 1.0
## 87MX4-5.5 1.924528 1.3279402 53 0.06432235   1   4   1   1 4.0
## 87MX5-1.7 1.534884 1.0082714 43 0.07141108   1   4   1   1 1.5
## 97CAT11.3 1.266667 0.8090398 45 0.06980613   1   4   1   1 1.0
## A-93      1.000000 0.0000000 54 0.06372398   1   1   1   1 1.0
## Allen3    1.000000 0.0000000 23 0.09764182   1   1   1   1 1.0
## Allen4    1.064516 0.3592106 31 0.08410445   1   3   1   1 1.0
## Apache    1.046875 0.3750000 64 0.05853422   1   4   1   1 1.0
## Baker     1.038462 0.2773501 52 0.06493788   1   3   1   1 1.0
## Burkett   1.025000 0.1581139 40 0.07404058   1   2   1   1 1.0
## Choctaw   1.000000 0.0000000 21 0.10218571   1   1   1   1 1.0
## Curtis    1.061224 0.4285714 49 0.06689625   1   4   1   1 1.0
## Elliott   1.148148 0.6266836 54 0.06372398   1   4   1   1 1.0
## Frutoso   1.000000 0.0000000 55 0.06314202   1   1   1   1 1.0
## Giles     1.000000 0.0000000 47 0.06830475   1   1   1   1 1.0
## Ideal     1.045455 0.3015113 44 0.07059492   1   3   1   1 1.0
## Major     1.000000 0.0000000 34 0.08030828   1   1   1   1 1.0
## Moore     1.052632 0.3244428 38 0.07596403   1   3   1   1 1.0
## Peruque   1.000000 0.0000000 21 0.10218571   1   1   1   1 1.0
## Riverside 1.015873 0.1259882 63 0.05899695   1   2   1   1 1.0
## SanFelipe 1.068182 0.4522670 44 0.07059492   1   4   1   1 1.0
## Shoshoni  1.019231 0.1386750 52 0.06493788   1   2   1   1 1.0
## Sioux     1.000000 0.0000000 42 0.07225621   1   1   1   1 1.0
## Stein     1.000000 0.0000000 46 0.06904320   1   1   1   1 1.0
## VC 1-68   1.068182 0.3339494 44 0.07059492   1   3   1   1 1.0
## Wichita   1.000000 0.0000000 44 0.07059492   1   1   1   1 1.0
##
## $comparison
## NULL
##
## $groups
##              TermBO groups
## 87MX4-5.5 1.924528      a
## 87MX5-1.7 1.534884      b
## 97CAT11.3 1.266667     bc
## Elliott   1.148148      c
## SanFelipe 1.068182      c
## VC 1-68   1.068182      c
## Allen4    1.064516      c
## Curtis    1.061224      c
## Moore     1.052632      c
## Apache    1.046875      c
```

```
## Ideal     1.045455      c
## Baker     1.038462      c
## 87MX1-1.2 1.037736      c
## Burkett   1.025000      c
## Shoshoni  1.019231      c
## Riverside 1.015873      c
## A-93      1.000000      c
## Allen3    1.000000      c
## Choctaw   1.000000      c
## Frutoso   1.000000      c
## Giles     1.000000      c
## Major     1.000000      c
## Peruque   1.000000      c
## Sioux     1.000000      c
## Stein     1.000000      c
## Wichita   1.000000      c
##
## attr(,"class")
## [1] "group"
```

```
tukey.test_5 <- TukeyHSD(aov5)

plot(tukey.test_5)
```

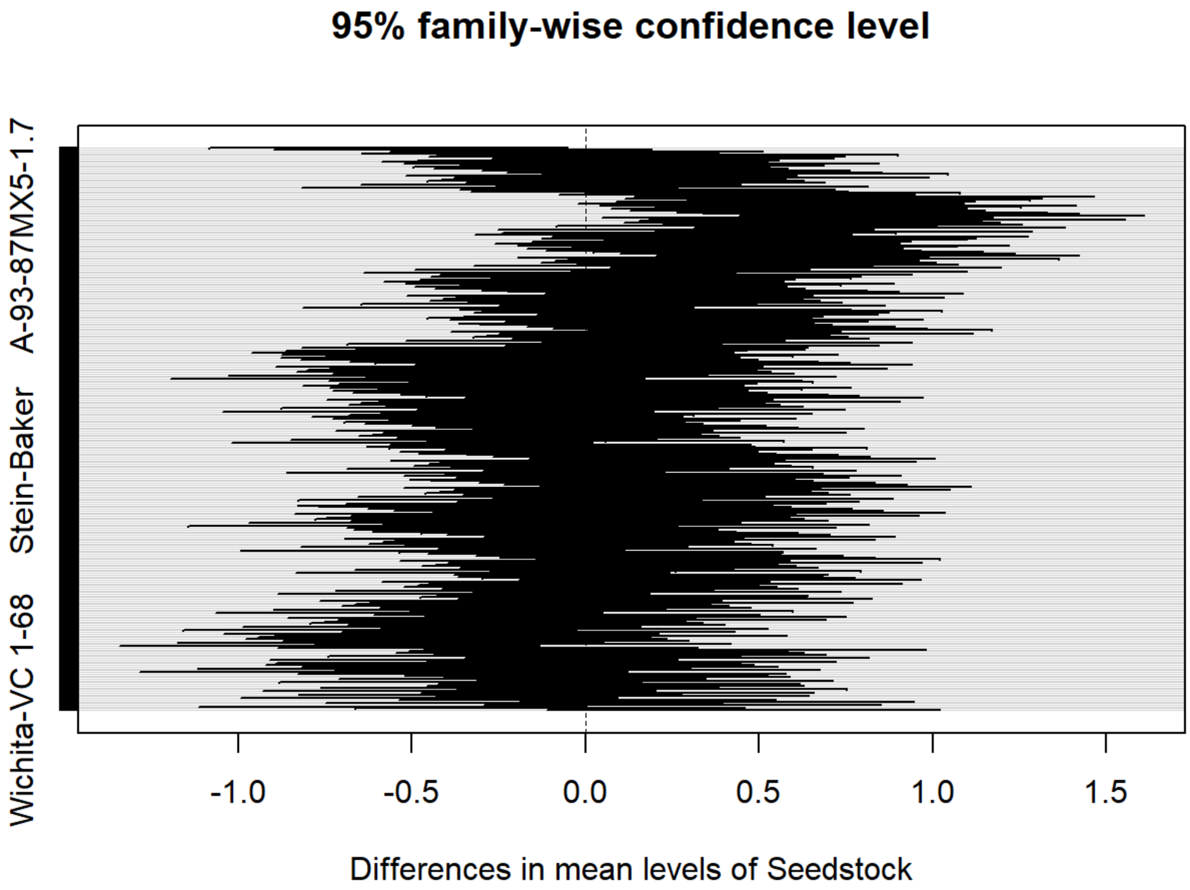
95% family-wise confidence level
Wichita-VC 1-68
Stein-Baker
A-93-87MX5-1.7
-1.0
-0.5
0.0
0.5
1.0
1.5
Differences in mean levels of Seedstock

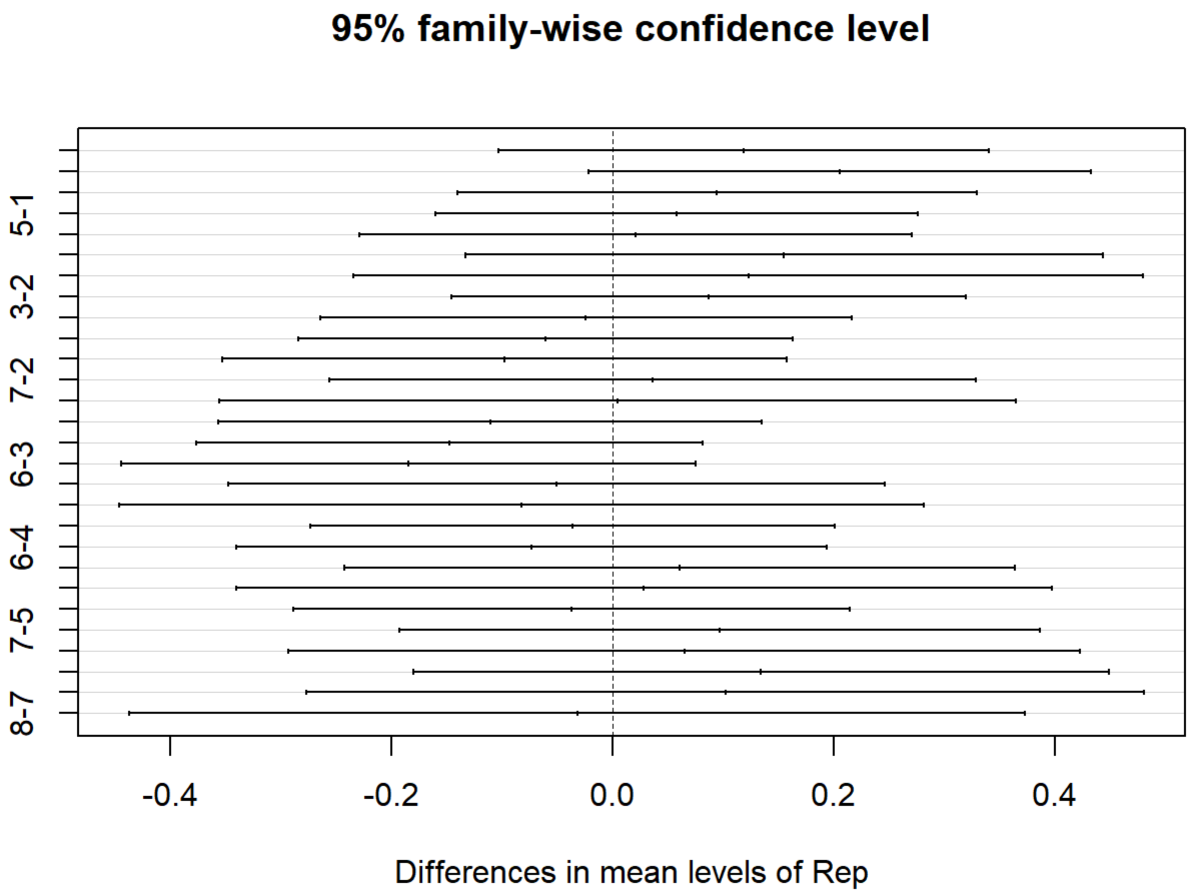
95% family-wise confidence level
5-1
3-2
7-2
6-3
6-4
7-5
8-7
-0.4
-0.2
0.0
0.2
0.4
Differences in mean levels of Rep

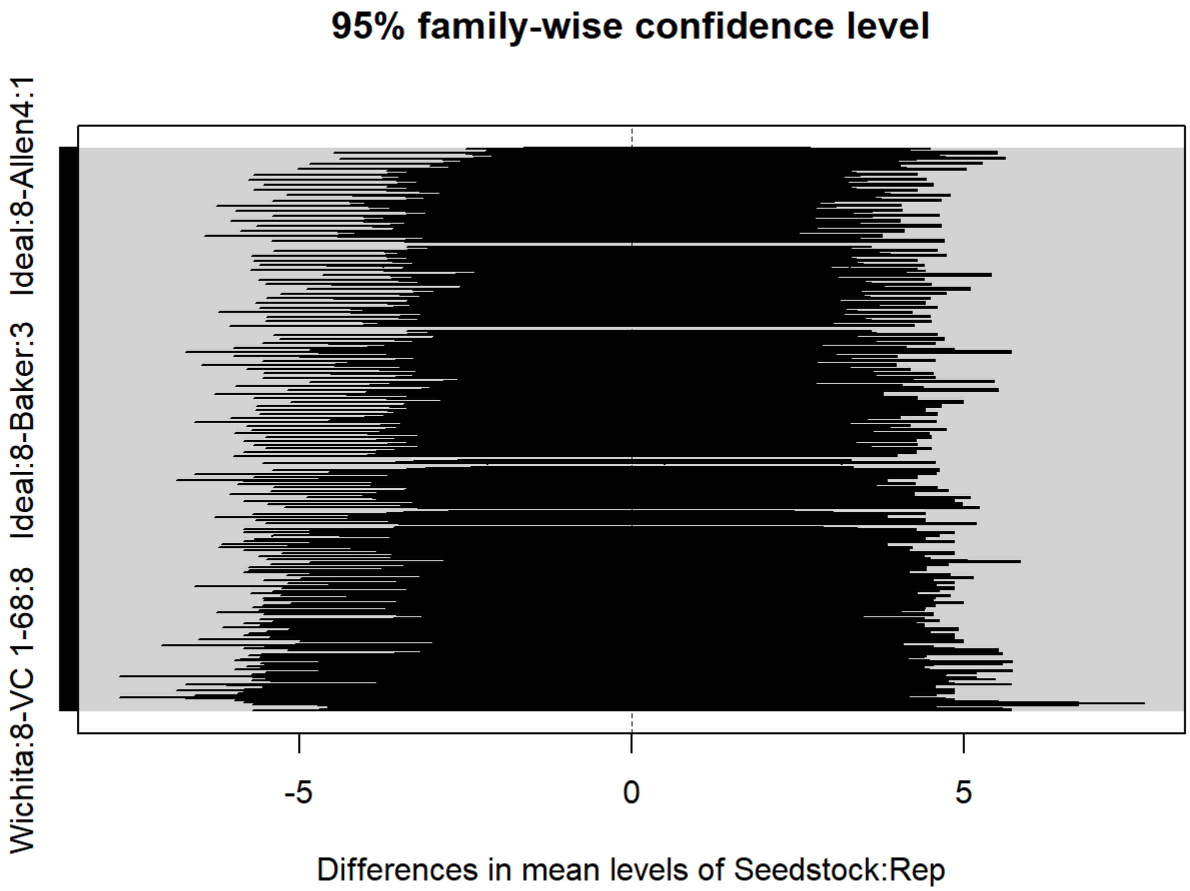


```
tukey.test_5 <- HSD.test(aov5, trt = 'Seedstock')
tukey.test_5
```

```
## $statistics
##     MSerror  Df     Mean       CV
##   0.5178489 967 3.032007 23.73402
##
## $parameters
##    test    name.t ntr StudentizedRange alpha
##   Tukey Seedstock  26         5.216432  0.05
##
## $means
##             ApWhrlO       std  r          se Min Max Q25 Q50 Q75
## 87MX1-1.2 2.981132 0.9300655 53 0.09884703   0   5   3   3   3
## 87MX4-5.5 2.415094 0.8645577 53 0.09884703   1   4   2   3   3
## 87MX5-1.7 2.627907 0.9764673 43 0.10974060   0   4   2   3   3
## 97CAT11.3 2.955556 0.7371800 45 0.10727420   1   5   3   3   3
## A-93      2.851852 0.8557774 54 0.09792750   0   4   2   3   3
## Allen3    3.217391 0.6712622 23 0.15005056   2   4   3   3   4
## Allen4    3.129032 0.8462441 31 0.12924707   0   4   3   3   4
## Apache    3.203125 0.6709313 64 0.08995215   0   5   3   3   3
## Baker     3.019231 0.6712699 52 0.09979295   2   5   3   3   3
## Burkett   2.950000 0.9044052 40 0.11378147   0   4   3   3   3
## Choctaw   3.142857 0.4780914 21 0.15703335   2   4   3   3   3
## Curtis    3.142857 0.6123724 49 0.10280246   2   4   3   3   4
## Elliott   3.000000 0.6729266 54 0.09792750   1   4   3   3   3
## Frutoso   3.054545 0.4875556 55 0.09703317   2   5   3   3   3
## Giles     3.212766 0.5874103 47 0.10496696   2   5   3   3   3
## Ideal     3.295455 0.7947369 44 0.10848638   2   5   3   3   4
## Major     3.441176 1.0207299 34 0.12341333   0   5   3   4   4
## Moore     3.026316 0.7161021 38 0.11673733   1   4   3   3   3
## Peruque   3.285714 0.9023778 21 0.15703335   0   4   3   3   4
## Riverside 3.063492 0.6188704 63 0.09066325   2   4   3   3   3
## SanFelipe 3.068182 0.4522670 44 0.10848638   2   4   3   3   3
## Shoshoni  3.153846 0.5381382 52 0.09979295   2   4   3   3   3
## Sioux     2.880952 0.8611501 42 0.11103935   0   4   3   3   3
## Stein     3.260870 0.8009656 46 0.10610177   2   5   3   3   4
## VC 1-68   2.704545 0.6317030 44 0.10848638   2   4   2   3   3
## Wichita   3.159091 0.5682769 44 0.10848638   2   5   3   3   3
##
## $comparison
## NULL
##
## $groups
##             ApWhrlO groups
## Major     3.441176      a
## Ideal     3.295455     ab
## Peruque   3.285714    abc
## Stein     3.260870    abc
## Allen3    3.217391    abc
## Giles     3.212766    abc
## Apache    3.203125    abc
## Wichita   3.159091    abc
## Shoshoni  3.153846    abc
## Choctaw   3.142857    abc
```

```
## Curtis    3.142857    abc
## Allen4    3.129032    abc
## SanFelipe 3.068182    abc
## Riverside 3.063492    abc
## Frutoso   3.054545    abc
## Moore     3.026316    abc
## Baker     3.019231    abc
## Elliott   3.000000    abc
## 87MX1-1.2 2.981132    abc
## 97CAT11.3 2.955556    abc
## Burkett   2.950000   abcd
## Sioux     2.880952   abcd
## A-93      2.851852    bcd
## VC 1-68   2.704545     cd
## 87MX5-1.7 2.627907     cd
## 87MX4-5.5 2.415094      d
##
## attr(,"class")
## [1] "group"
```

Note that the `echo = FALSE` parameter was added to the code chunk to prevent printing of the R code that generated the plot.
