# Supplementary figures and images for: Phenotypic diversity and population structure of Pecan (Carya illinoinensis) collections reveals geographic patterns
Source: Sci Rep. 2024 Aug 10;14:18592. doi: 10.1038/s41598-024-69521-1 (PMC11316781; doi:10.1038/s41598-024-69521-1)

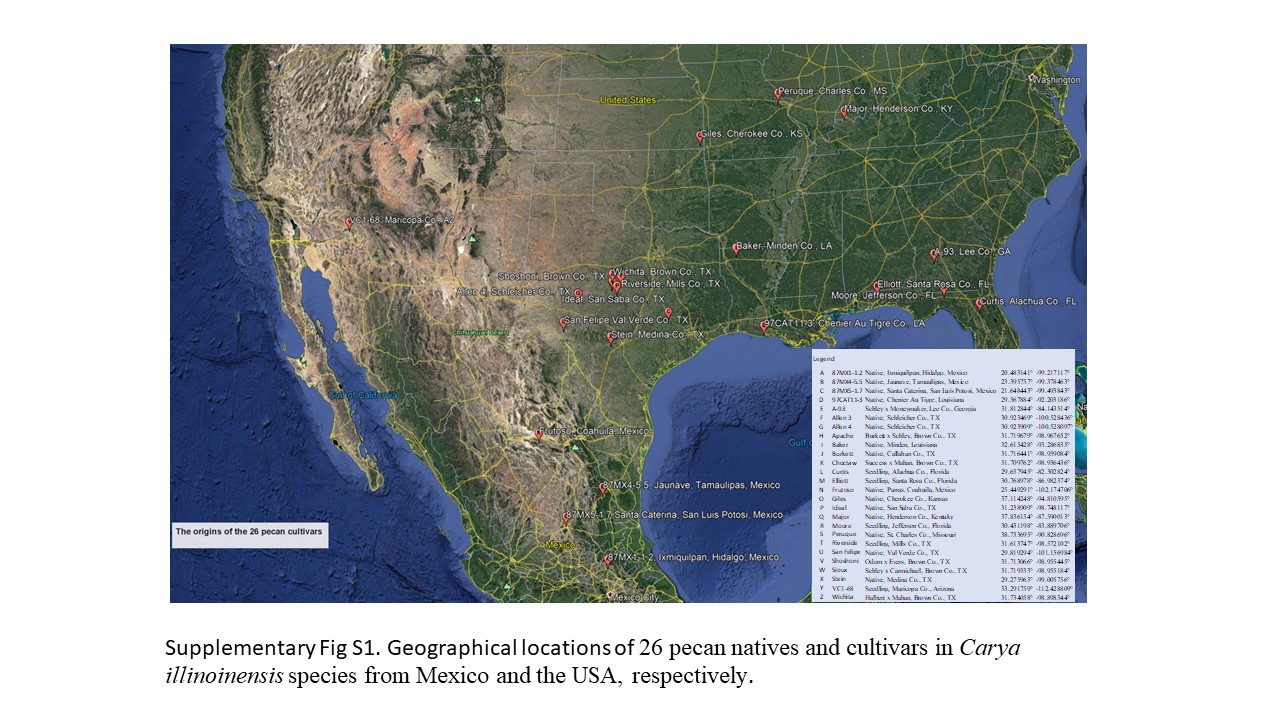

Supplement: Supplementary file 1 — Supplementary Information. [file 41598_2024_69521_MOESM1_ESM.zip › Fig S1_R, 2024.jpg]

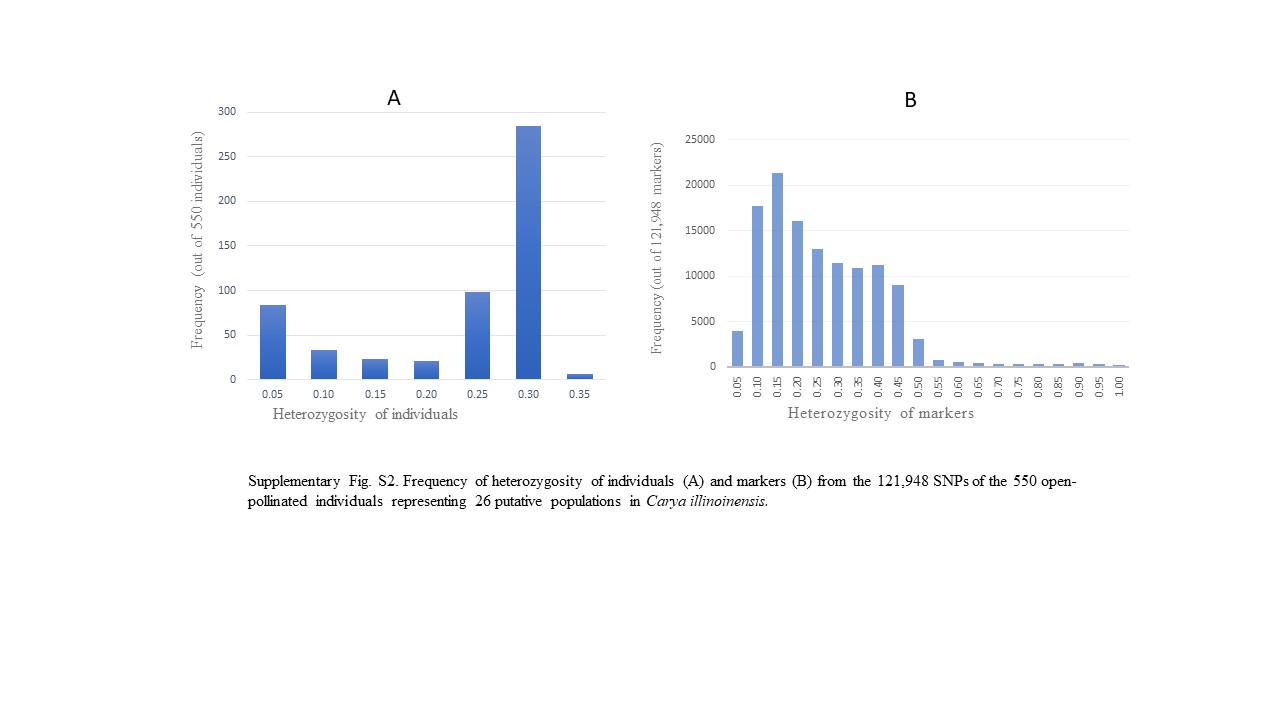

Supplement: Supplementary file 1 — Supplementary Information. [file 41598_2024_69521_MOESM1_ESM.zip › Fig S2_R, 2024.jpg]

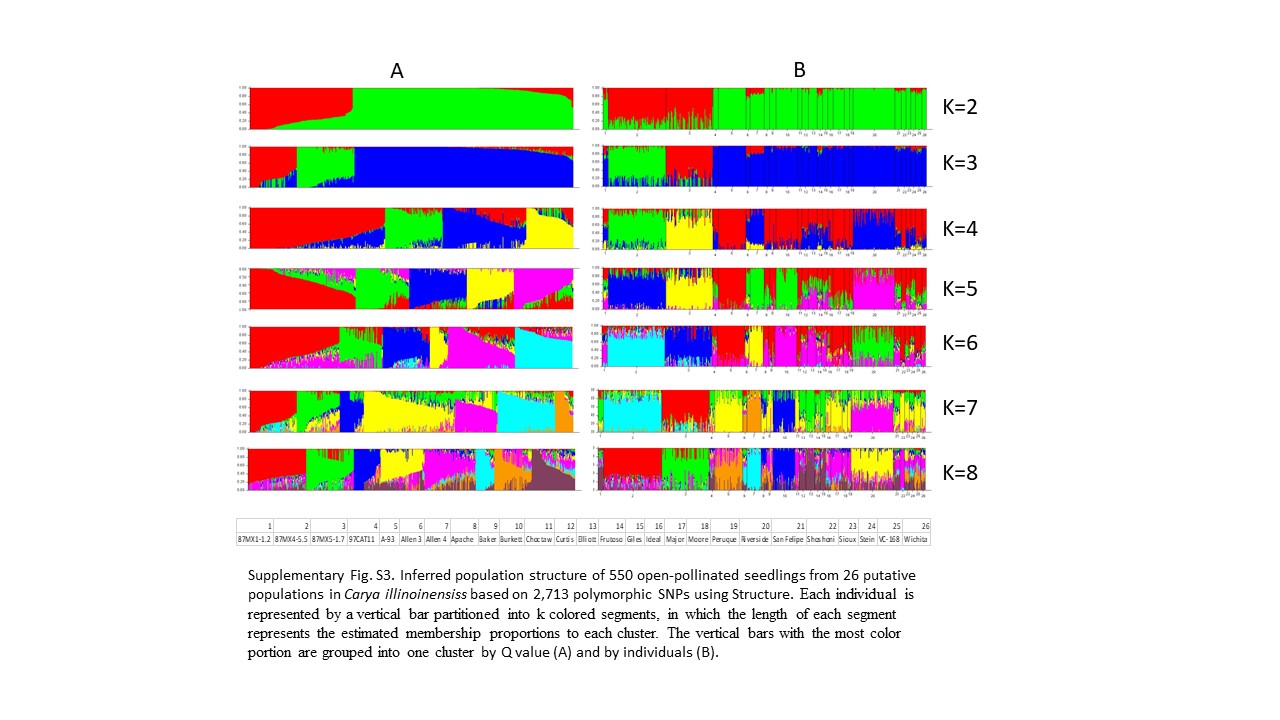

Supplement: Supplementary file 1 — Supplementary Information. [file 41598_2024_69521_MOESM1_ESM.zip › Fig S3_R, 2024.jpg]

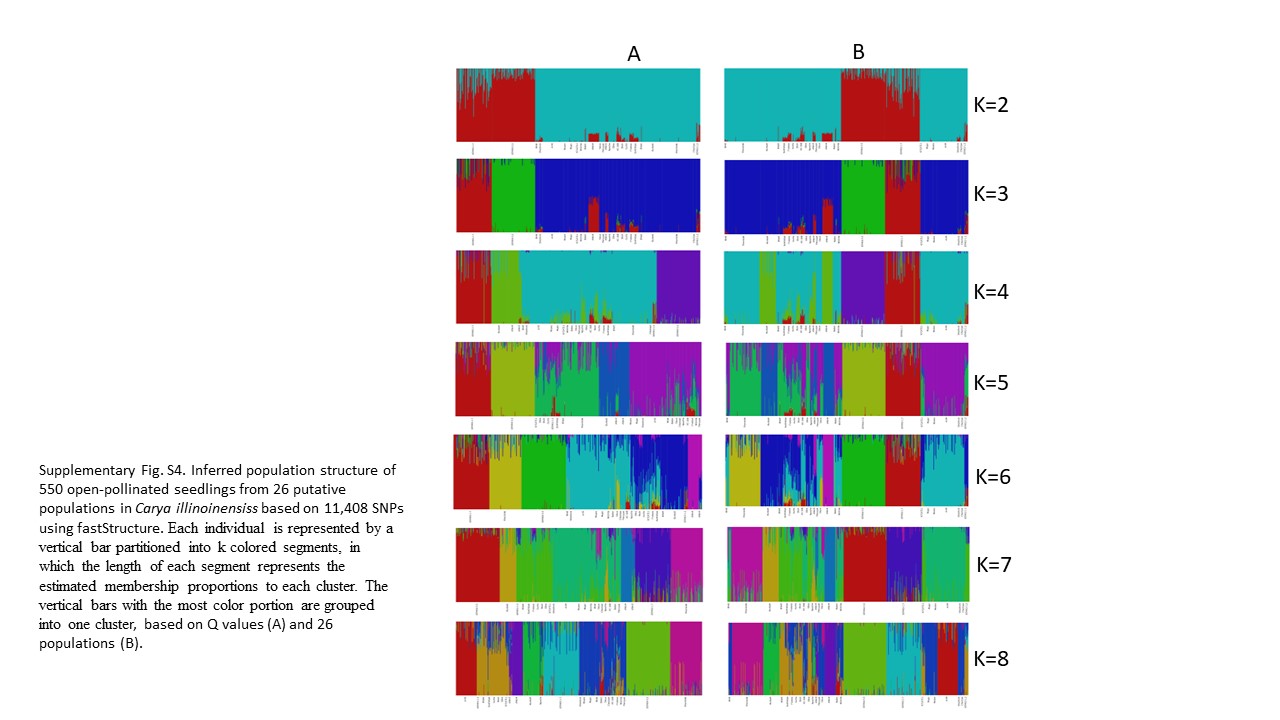

Supplement: Supplementary file 1 — Supplementary Information. [file 41598_2024_69521_MOESM1_ESM.zip › Fig S4_R, 2024.jpg]

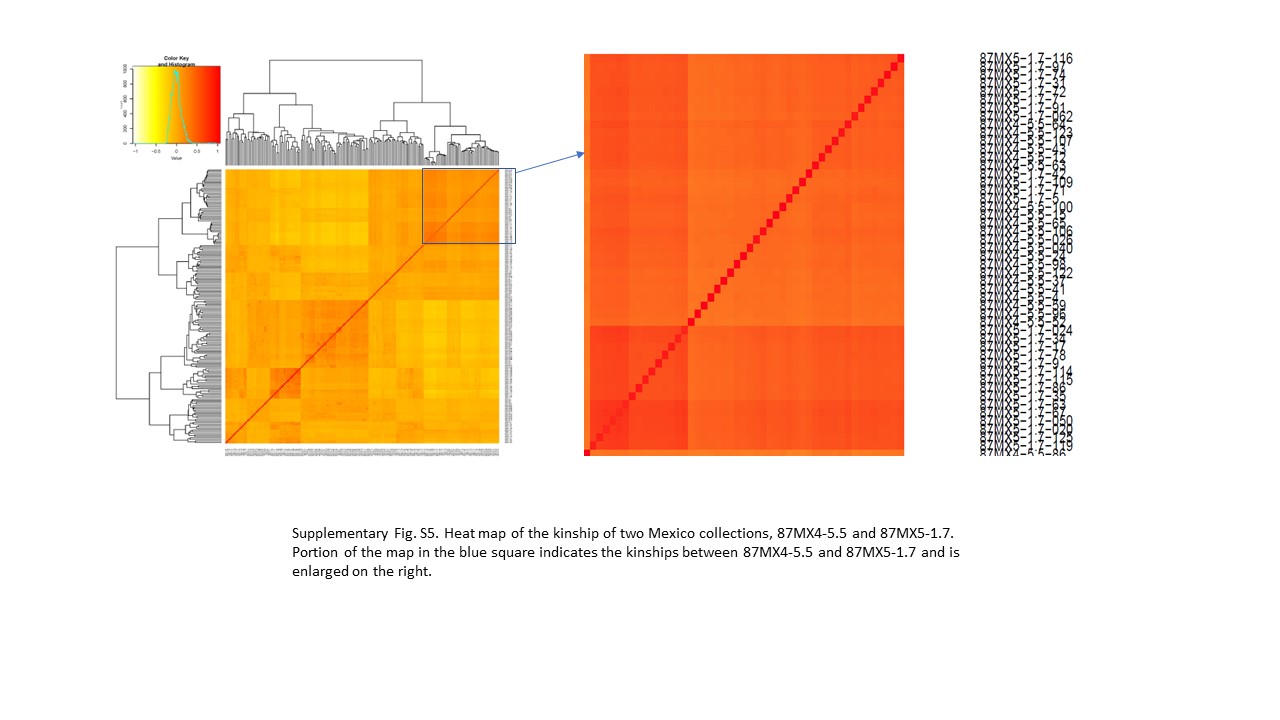

Supplement: Supplementary file 1 — Supplementary Information. [file 41598_2024_69521_MOESM1_ESM.zip › Fig S5_R, 2024.jpg]

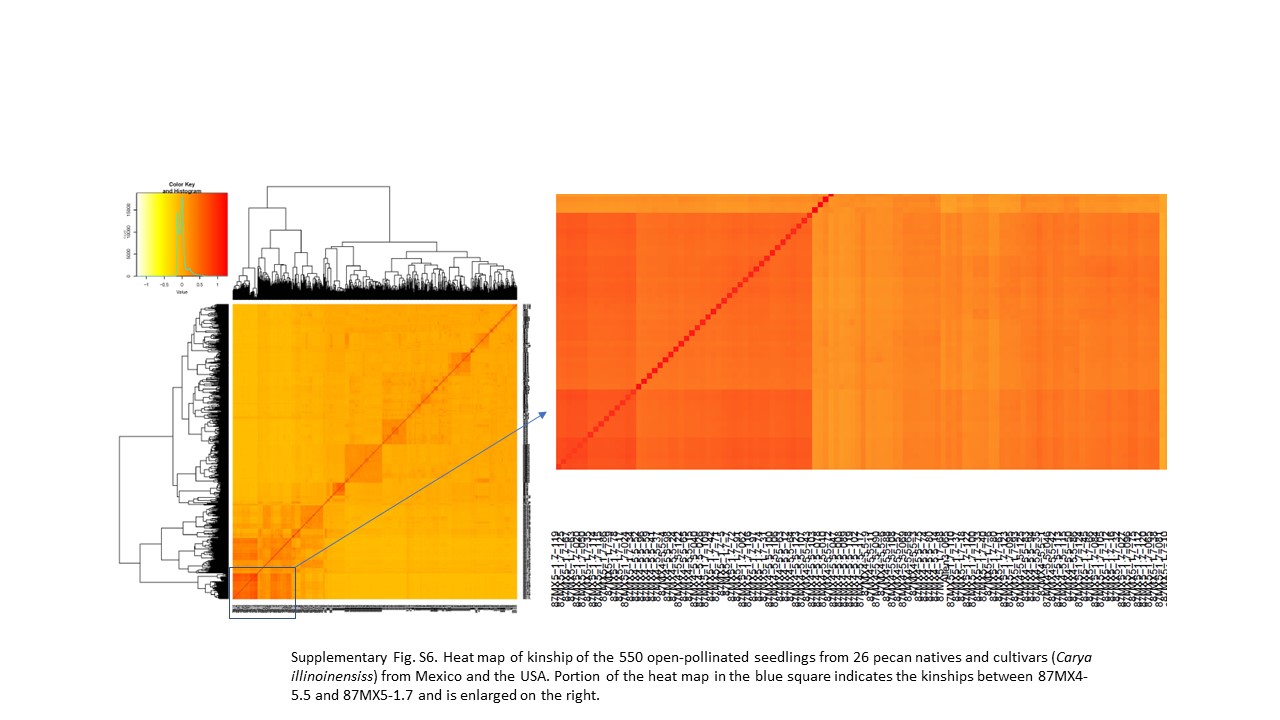

Supplement: Supplementary file 1 — Supplementary Information. [file 41598_2024_69521_MOESM1_ESM.zip › Fig S6_R, 2024.jpg]
